# The Spectrum of Mitochondrial Ultrastructural Defects in Mitochondrial Myopathy

**DOI:** 10.1038/srep30610

**Published:** 2016-08-10

**Authors:** Amy E. Vincent, Yi Shiau Ng, Kathryn White, Tracey Davey, Carmen Mannella, Gavin Falkous, Catherine Feeney, Andrew M. Schaefer, Robert McFarland, Grainne S. Gorman, Robert W. Taylor, Doug M. Turnbull, Martin Picard

**Affiliations:** 1 Wellcome Trust Centre for Mitochondrial Research, Institute of Neurosciences, Newcastle University, Newcastle upon Tyne, UK; 2 EM Research Services, Newcastle University, Newcastle upon Tyne, UK; 3 Wadsworth Center, New York State Department of Health, Albany, NY, USA; 4 Department of Psychiatry, Division of Behavioral Medicine, Columbia University Medical Center, New York, NY, USA; 5 Department of Neurology and Columbia Translational Neuroscience Initiative, H. Houston Merritt Center, Columbia University Medical Center, New York, NY, USA

## Abstract

Mitochondrial functions are intrinsically linked to their morphology and membrane ultrastructure. Characterizing abnormal mitochondrial structural features may thus provide insight into the underlying pathogenesis of inherited and acquired mitochondrial diseases. Following a systematic literature review on ultrastructural defects in mitochondrial myopathy, we investigated skeletal muscle biopsies from seven subjects with genetically defined mtDNA mutations. Mitochondrial ultrastructure and morphology were characterized using two complimentary approaches: transmission electron microscopy (TEM) and serial block face scanning EM (SBF-SEM) with 3D reconstruction. Six ultrastructural abnormalities were identified including i) paracrystalline inclusions, ii) linearization of cristae and abnormal angular features, iii) concentric layering of cristae membranes, iv) matrix compartmentalization, v) nanotunelling, and vi) donut-shaped mitochondria. In light of recent molecular advances in mitochondrial biology, these findings reveal novel aspects of mitochondrial ultrastructure and morphology in human tissues with implications for understanding the mechanisms linking mitochondrial dysfunction to disease.

Skeletal muscle fibers contain large numbers of mitochondria classically known for their role in ATP synthesis through oxidative phosphorylation (OXPHOS), which fuels the energy-demanding process of muscle contraction^1^,^2^. However, research in recent decades has revealed that beyond energy production mitochondria also perform a number of additional functions. They contribute to Ca^2+^ homeostasis^3^,^4^ and redox signalling^5^,^6^, release pro-apoptotic factors regulating cell death^7^, synthesize essential macromolecules including heme molecules^8^, regulate nuclear gene expression^9^,^10^, and can release immunogenic pro-inflammatory molecules into the cytoplasm and systemic circulation^11^. Beyond expanding our fundamental understanding of mitochondrial biology^12^, these advances have challenged the commonly reported view of mitochondria as a cellular powerhouse and thus revealed new cellular mechanisms whereby mitochondrial dysfunction causes clinical disease^13^,^14^,^15^,^16^.

Of particular interest to neuromuscular disorders is the link between mitochondrial ultrastructure and function^17^,^18^,^19^. Abnormal mitochondrial ultrastructure, particularly the highly conserved inner mitochondrial membrane (IMM) cristae where a number of OXPHOS enzymes reside, is linked to altered mitochondrial function and signalling that may contribute to pathophysiology (e.g., ref. 20). Furthermore, the molecular machinery that regulates mitochondrial morphology and ultrastructural transitions has in part been defined and experimental manipulation of various components in model systems has reinforced causal links between mitochondrial structures and functions^21^. Thus, the determinants of mitochondrial ultrastructure and its functional bearings have implications for understanding the pathophysiology in human mitochondrial disease^22^. However, mitochondrial ultrastructural defects in mitochondrial myopathy have not been thoroughly examined in light of recent molecular advances in mitochondrial biology.

The first description of mitochondrial structures in human skeletal muscle using electron microscopy appeared in the 1960s^23^,^24^,^25^. Abnormal skeletal muscle mitochondrial morphology and ultrastructure has since been reported in the context of aging and several neuromuscular disorders, but most previous studies involved only a limited spectrum of defects and/or poorly-defined genetic disorders (e.g., refs 26, 27 see Table S1). Previous investigations of mitochondrial ultrastructure in clinical samples were also limited to standard (single-plane) transmission electron microscopy (TEM), precluding analysis of complex three-dimensional mitochondrial structures of potential functional significance.

Here, we first conducted a systematic review of the literature to document mitochondrial ultrastructural abnormalities in skeletal muscle of individuals with mitochondrial disease published to date. We then combined two electron microscopy methods: TEM and serial block face scanning electron microscopy (SBF-SEM) to examine muscle biopsies from individuals with molecularly defined mitochondrial DNA (mtDNA) mutations. This in depth re-examination of skeletal muscle mitochondrial ultrastructure in light of recent advances in mitochondrial biology reveals abnormal features not previously reported in human tissues, thus expanding the spectrum of mitochondrial EM pathology in mitochondrial disease.

## Results

### Systematic review of the literature

We first performed a systematic review of the literature that identified 135 publications reporting EM findings in skeletal muscle biopsies of individuals with mitochondrial disease. These are summarized in Table S1, including information about the muscle biopsied, subject ages, clinical phenotype, and genetic diagnosis where available. Among the most commonly reported features were enlarged mitochondrial size with swelling, paracrystalline inclusions (PCIs), and electron-dense precipitates in the mitochondrial matrix. This systematic review indicated that most articles were case studies, published prior to technological progress enabling genetic diagnosis, or prior to recent discoveries of the molecular aspects of mitochondrial ultrastructure, thus limiting the interpretation of these observations.

### Normal mitochondrial ultrastructure

**Figure 1:**
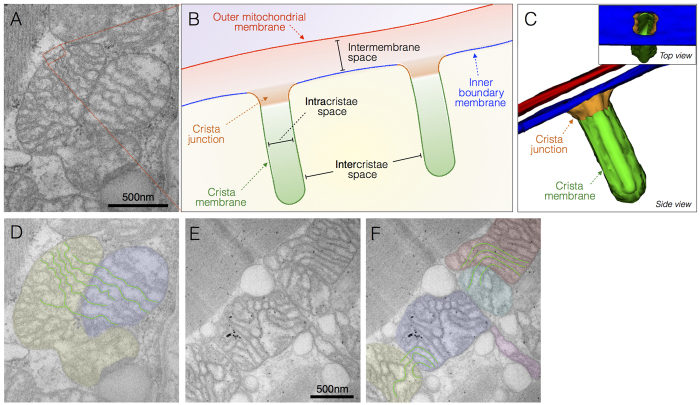
Normal mitochondrial ultrastructure. (**A**) Transmission electron micrograph of normal mitochondria in human skeletal muscle showing typical tubular cristae, and crista junctions. (**B**) Schematic representation of crista junctions, associated structures, and parameters measured in this study. (**C**) Three-dimensional schematic of normal tubular cristae structure. The outer mitochondrial membrane (OMM, red), and three functionally distinct regions of the inner mitochondrial membrane (IMM): inner boundary membrane (blue), crista junction (orange), and crista membrane (green). (**D**) Same image as in A with pseudocolored adjacent but distinct mitochondria and outlined crisate membranes undergoing trans-mitochondrial cristae coordination (see ref. 34 and text for discussion). (**E**) Unprocessed and (**F**) pseudocolored transmission electron micrographs of normal human skeletal muscle illustrating mitochondria with normal shapes and sizes, with electron-dense curvilinear cristae, with some exhibiting trans-mitochondrial cristae coordination.

Parameters for normal mitochondrial ultrastructure are established (e.g., refs 28, 29, 30) and were confirmed in biopsies from two individuals with normal skeletal muscle mitochondrial histochemistry. Mitochondria are double membrane organelles with the inner (IMM) and outer mitochondrial membranes (OMM) surrounding the mitochondrial matrix and intermembrane space, respectively (Fig. 1). The convoluted IMM bends inwards^31^,^32^ and can be divided into three regions; inner boundary membrane, the crista junction, and the cristae membrane (Fig. 1B,C)^33^. In some cases where mitochondria are tethered by an inter-mitochondrial junction (IMJ), trans-mitochondrial cristae coordination between distinct organelles can be observed^34^ (Fig. 1A,D). Also in normal mitochondria, the matrix is a single continuous space that contains enzymes involved in biochemical transformations and biosynthetic reactions, as well as several copies of the mitochondrial genome. Although frequently depicted as ovoid or bean-shaped, mitochondria oscillate between tubular and spherical morphology determined by highly dynamic fusion and fission processes, which also determine their sizes^21^.

Here, we aimed to document deviations from normal mitochondrial ultrastructure in molecularly-defined cases of mitochondrial disease. We examined muscle biopsies from seven patients who harboured a primary mtDNA mutation: single large-scale mtDNA deletion (n = 3), *m.8344A>G tRNA*^*Lys*^ (n = 3), or *m.3243A>G tRNA*^*Leu(UUR)*^ (n = 1). Six mitochondrial ultrastructural abnormalities were identified as candidate features linked to known myopathic mechanisms. Demographic, genetic, biochemical, and clinical information for each subject is provided in Table 1. A summary of ultrastructural findings for all subjects is provided in Table 2. Each observed feature is first presented below, and their potential biological and clinical significance subsequently discussed in light of recent advanced in mitochondrial biology.

### Paracrystalline inclusions (PCIs)

**Figure 2:**
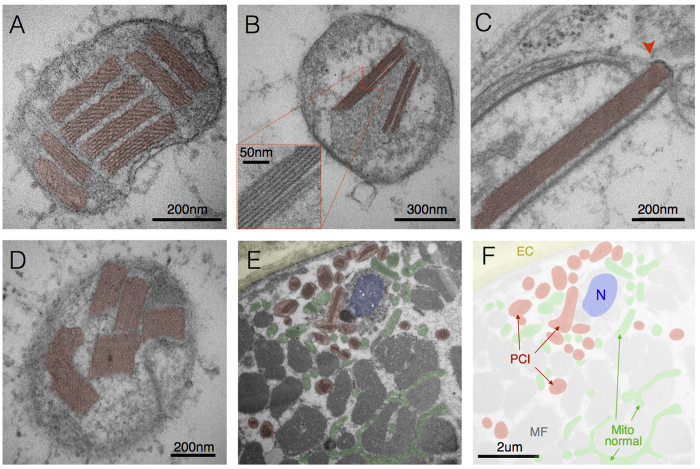
Paracrystalline inclusions (PCIs). (**A**) Type I PCIs occupying most of a mitochondrion’s volume in skeletal muscle with a single mtDNA deletion (patient 1). (**B**) Linear Type I PCI in mitochondrion from a case of m.8344A>G (patient 5). (**C**) Disruption of IMM and OMM (arrowhead) by a rigid type II PCI, and (**D**) other examples of type II PCIs from a case of *m.8344A>G* (patient 4). (**E**) Subsarcolemmal region of a muscle fiber showing the extracellular space (EC, yellow) and sarcolemma, profile of a nucleus (N, blue), myofibrils (MF), mitochondria with PCI (red) and normal mitochondria (green), with (**F**) pseudocolored mask (patient 1). Note the greater abundance of PCI-containing mitochondria in the perinuclear SS compartment compared to the intermyofibrillar compartment, consistent with the distinctive requirement for *de novo* protein synthesis for PCI formation.

PCIs were observed in a patient harbouring a single, large-scale mtDNA deletion (patient 1) and chronic progressive external opthalmoplegia (CPEO) and in two patients manifesting with myopathy with the *m.8344A>G* mutation (patients 4 and 5). PCIs present as rigid rectangular crystals of approximately 250 nm in length and 50 nm in width. They consist of stacked sheets that either run obliquely (Fig. 2A,D) or parallel (Fig. 2B,C) relative to the length of the paracrystal. Their rigidity is evidenced by their occasional deformation or rupture of membranes (Fig. 2C). As for the other defects discussed below, substantial heterogeneity existed in PCI distribution even within single cells, with some mitochondria exhibiting normal ultrastructure, and other containing PCIs of various types and sizes. Although PCIs were observed in both subsarcolemmal (SS, beneath the plasma membrane and near nuclei) and intermyofibrillar (IMF, between myofibrils) compartments, PCIs were generally more prevalent in SS mitochondria (Fig. 2E,F).

PCIs are classified in two major types characterized by defined structural pattern, size, and location within the mitochondria^35^. Both type-I PCIs comprised of stacked sheets with discernible structure forming rectangular inclusions in the intracristae space (Fig. 2A,B) and type-II PCIs with electron dense substructure and residing in the intracristae and intermembrane space (Fig. 2D) were observed here. Consistent with previous observations^35^, we found type-I and type-II PCIs to be mutually exclusive within individual patients; type-I PCIs were identified in patients 1 and 5, whereas type-II PCIs were observed in patient 4.

### Cristae linearization and angular features

**Figure 3:**
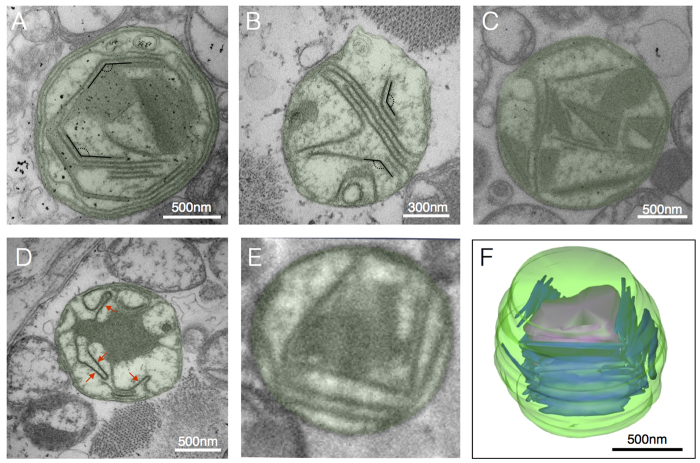
Linearization and geometrical cristae features. (**A,B**) Enlarged mitochondria containing linearized cristae juxtaposed at nearly identical angles, and (**C**) linearized cristae forming various geometrical shapes, and (**D**) linearized IMM segments (arrows) joined by curved segments from a case of *m.8344A>G* (patient 4). **(E)** SBF-SEM image with example of linearized cristae and geometrical shapes and **(F)** three-dimensional reconstruction of mitochondrion in E (patient 4).

In contrast to the normal invaginations of the IMM (Fig. 1), we observed linearization of cristae membranes and abnormal angular (i.e., geometrical) features in patient 4 (Fig. 3). These structures included rigid linear cristae juxtaposed at angles of 124.7 ± 7.4° (Fig. 3A,B), often in association with electron dense inclusions (Fig. 3C,D). Linear cristae segments show enhanced electron density, suggesting the presence of large molecular weight proteins and/or substantial change in membrane lipid composition. Three-dimensional reconstruction from SBF-SEM data demonstrated the organization of linearized electron-dense cristae as continuous sheets encompassing cargo, rather than isolated tubular structures (Fig. 3E,F).

### Concentric cristae or “onion-like” mitochondria

**Figure 4:**
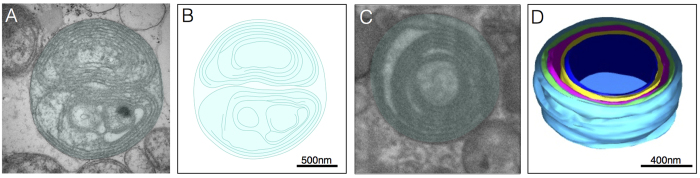
Concentric “onion-shaped” cristae. (**A**) Multiple overlaid layers of double OMM/IMM membranes forming two major compartments in a case of the *m.8344A>G* (patient 4), and (**B**) two-dimensional reconstruction of membrane structures. (**C**) Example of concentric cristae compartments (patient 4) imaged with SBF-SEM, with (**D**) corresponding three-dimensional reconstruction.

In normal mitochondria the inner boundary membrane periodically invaginates into tubular or fenestrated laminar cristae, with the resulting crista junctions forming pores between the intermembrane and intracristae spaces (Fig. 1). Contrary to this pattern, in TEM from patients 4 and 6 with the *m.8344A>G* mutation we observed several mitochondria devoid of normal cristae and crista junctions (CJs), but with abnormal “onion-like” concentric membranes free of fenestration (Fig. 4), also previously referred to as “tubular parallel cristae” or “concentric laminated bodies” (see Table S1). Three-dimensional reconstructions confirmed the tight layering of discontinuous concentric membranes sheets (Fig. 4C,D).

The intermembrane space separating the OMM-IMM was similar between mitochondria with concentric (7.1 ± 1.6 nm) and normal cristae (7.9 ± 1.2 nm, *P* = 0.45). However, the width of the intracristae spaces (Fig. 1B) was reduced by approximately 15% in mitochondria with concentric cristae (7.78 ± 1.0 nm) compared to normal mitochondria (9.20 ± 1.0 nm, *P* < 0.05), indicating a tightening of cristae membranes in onion-like mitochondria. Furthermore, as expected the distance between two adjacent cristae (Fig. 1B, intercristae space) was substantially smaller in mitochondria with concentric cristae (22.5 ± 4.5 nm) than in normal mitochondria (60.2 ± 9.9 nm, *P* < 0.001), indicating denser packaging of membranes in dysfunctional mitochondria.

### Compartmentalisation

**Figure 5:**
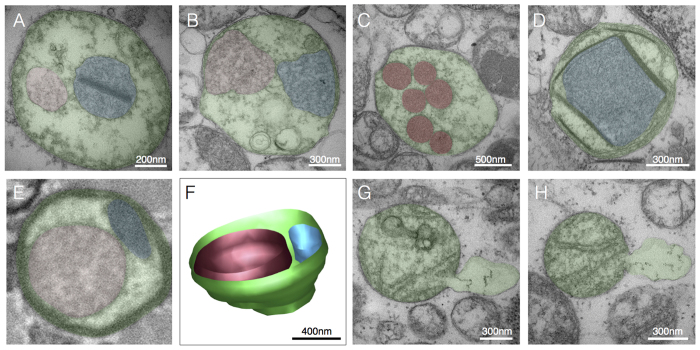
Compartmentalization. (**A**) Example of membrane-bound sub-mitochondrial compartments located centrally and (**B**) peripherally in contact with the inner boundary membrane, from cases of *m.8344A>G* mutation (patient 5) and single mtDNA deletion (patient 3), respectively. (**C**) Electron-dense round compartments distributed in the mitochondrial matrix in a case of single mtDNA deletion (patient 3). (**D**) Compartment bound by linearized electron-dense membranes in a case of *m.8344A>G* (patient 4). (**E**) Cross-sectional image of a mitochondrion with two distinct compartments of different electron density, and (**F**) three-dimensional reconstruction from SBF-SEM. (**G,H**) Examples of OMM protrusion and distension consistent with the release of mitochondrial components in the cytoplasm in a case of *m.8344A>G* mutation (patient 4). Pseudocolored areas indicate the major compartment bound by the OMM (green), and sub-compartments (red and blue).

Normal mitochondria consist of 2 compartments: a single mitochondrial matrix compartment, and a contiguous intermembrane/intracristae space. We observed an increased number of compartments per mitochondrion particularly in patient 3 with a single, large-scale mtDNA deletion and CPEO, and patient 5 with a *m.8344A>G* mutation and mild myopathy. Abnormal compartments were bound by a single or double membrane with distinct molecular or ionic composition evidenced by differences in electron density between each compartment (Fig. 5A,B), dense amorphous material (Fig. 5C), occasionally bounded by electron-dense linearized membranes (Fig. 5D). Notably, compartmentalization appears to involve the complete absence of crista junction and normal cristae structures. Three-dimensional reconstruction of mitochondria exhibiting apparent compartmentalization in cross-section confirmed the complete spatial isolation of individual compartments without connection to the outer boundary membrane (Fig. 5E,F). Localized distension of the OMM with apparent release of mitochondrial matrix content into the cytoplasm was also observed (Fig. 4G,H).

### Nanotunneling

**Figure 6:**
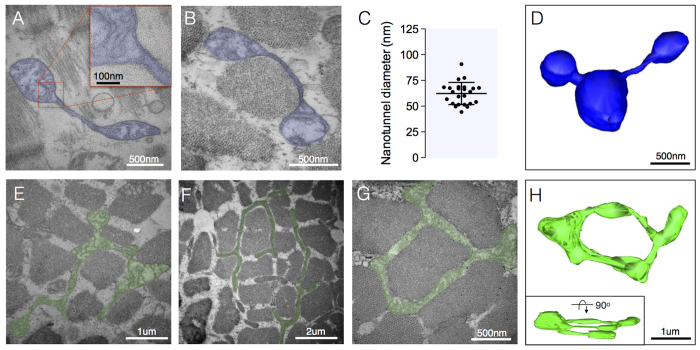
Nanotunelling and hyperbranching. (**A,B**) Examples of mitochondrial nanotunnels composed of both OMM and IMM, but devoid of cristae in a case of *m.3243A>G* (patient 7). (**C**) Variation in nanotunnel diameter, with each datapoint representing the average of the smallest and largest diameters measured for individual nanotunnels across all subjects (n = 26). (**D**) Three-dimensional reconstruction of three mitochondria connected via nanotunnels from a case of *m.3242A>G* (patient 7). (**E,F**) Examples of highly branched mitochondria (hyperfusion) from cases of *m.3243A>G* (patient 7) (**G**) Donut-shaped mitochondrion denoting self-fusion from a case of single mtDNA deletion (patient 2). (**H**) Three-dimensional reconstruction of a donut-shaped mitochondrion from a case of *m.8344A>G* (patient 6).

We observed mitochondrial nanotunnels (Fig. 6A,B) in three of the seven patients (patients 2, 3 and 7). The average diameter of nanotunnels was 62.3 ± 10.7 nm (Fig. 6C) and was similar between patients. Three-dimensional reconstructions further demonstrated that nanotunnels consist of thin tubular projections extending from one or more mitochondria. The nanotunnel length averaged 623 nm, ranging from 206 nm to 2.3 μm (Fig. 6D).

### Hyperbranching and donut mitochondria

Extensively branched mitochondria were particularly abundant in a case of *m.3243A>G* with 21% heteroplasmy (patient 7) (Fig. 6E,F). Interestingly, donut-shaped mitochondria arising from self-fusion of a mitochondrion were also observed in cases single mtDNA deletion at 34% heteroplasmy (patient 2) and *m.8344A>G* at 40% heteroplasmy (patient 6) (Fig. 6G,H). Three-dimensional reconstruction confirmed the unique toroid nature of donut mitochondria (Fig. 6H).

## Discussion

Mitochondrial biology is replete with examples where mitochondrial ultrastructure is mechanistically linked to functions^17^,^18^, and vice versa. In an attempt to understand the pathogenesis of mitochondrial disease, early electron microscopy investigations identified common ultrastructural abnormalities such as swelling and PCIs. However, most observations were mainly made prior to the identification of molecular determinants of mitochondrial structure and function (Table S1), thus limiting the spectrum of conceivable observations and the interpretation of their mechanistic significance. Improved diagnosis enable the identification of pathogenic mutations in an unprecedentedly large proportion of patients^36^. Here, building from recent advances in mitochondrial biology imaging technology, and genetic diagnosis, we examined the spectrum of mitochondrial ultrastructural features in a clinically characterized group of patients with genetically defined mitochondrial disease.

The literature to date characterizes what constitutes normal mitochondrial morphology in human and mouse skeletal muscle, including the organization of the IMM into cristae (Fig. 1). Using our electron microscopy approaches, we documented comparable normal mitochondrial morphology in two individuals with normal skeletal muscle mitochondrial function^34^, confirming that our biopsy and imaging methods preserved normal mitochondrial ultrastructure. Normal ultrastructure notably included the physical coordination of cristae between pairs of distinct mitochondria linked by an inter-mitochondrial junction (Fig. 1A,D), a phenomenon which has been shown to be resistant to genetic disruption of respiratory chain function in mice, independent of mitofusins, evolutionary conserved from mollusk to mammals, and inducible to some extent by the physical rapprochement of energized mitochondria *in vitro*^34^. In general, from a methodological perspective, it must be noted that consistent with the mosaic distribution of affected and non-affected cells in mitochondrial myopathy, substantial heterogeneity and mosaicism in mitochondrial ultrastructure exists between individuals, between muscle fibers, and even within single cells (Fig. S1).

Overall, the electron microscopic survey described here expands the spectrum of mitochondrial ultrastructural abnormalities known to exist in human. Some previously reported features are non-specific pathological hallmarks of mitochondrial myopathy such as PCIs, enlarged mitochondria, concentric “onion-like” cristae. Other features not previously reported include linearization and angular arrangement of cristae, localized membrane distension, nanotunnels, and donut-shaped mitochondria. These features are discussed below in context of recent findings in mitochondrial biology, with an emphasis on their potential significance to mitochondrial function and pathology.

### Paracrystalline inclusions

Although sample size was small, type-I (n = 2) and type-II (n = 1) PCIs were found to be mutually exclusive similar to previous reports^35^. The cause of this mutual exclusivity is, to date, unknown. Notably, two related patients with the *m.8344A>G* mutation (mother and daughter, patients 4 and 5) manifested different PCIs, indicating that factors other than the mtDNA mutation and nuclear background likely interact to determine disease phenotype. PCI analysis in larger cohorts of patients would be required to understand the specific conditions leading to different types of PCIs. However, it is worth noting that in our study occurrence of type II PCIs correlated clinically with most severe cytochrome c oxidase deficiency and lactic acidosis, and structurally with highest occurrence of concentric and linearized inner membranes.

We further note that PCIs appear more commonly in subsarcolemmal mitochondria. PCIs are composed of crystallized mitochondrial creatine kinase (CK)^37^, likely as a result of a compensatory upregulation of CK due to a deficiency of creatine phosphate shuttle activity. Reduced shuttle activity likely results from reduced ATP synthesis secondary to OXPHOS defects, leading to CK protein accumulation and PCI formation. Thus, contrary to most known pathognomonic processes triggered by loss-of-function mutations (i.e., absence of a protein or functional mutation), PCI formation involves the accumulation of a functional protein. Protein accumulation requires *de novo* synthesis, which depends upon upregulation of the CK gene in the nuclear compartment, and local translation of the gene product. This molecular requirement for PCI could explain the preferential abundance in the SS mitochondria that are inherently closer to myonuclei than IMF mitochondria.

### Linearized cristae membranes

Linearized cristae membranes with altered electron density and a rigid angular appearance were observed in a single patient (patient 4). These were commonly found in association with PCIs and changes to both membrane and matrix electron density. Altered protein composition or supramolecular assembly of protein complexes, particularly of the ATP synthase, which is heavily involved in determining IMM folding^32^,^38^, influence membrane curvature. Changes in the fluidity or rigidity of the IMM could also be caused by altered cardiolipin content in the IMM^39^,^40^,^41^. Interestingly, we noted linearized cristae membranes juxtaposed at an angle of approximately 120**°**. Domènech *et al*. also reported hexagonal structures with membranes exhibiting 120° angles in cardiolipin-containing phospholipid bilayer^42^, suggesting that cristae linearization and abnormal angular features in mitochondrial myopathy could result from altered membrane lipid composition. The sole case with prominent linearized inner membranes in our study (patient 4) was concurrent with the only example of type II PCIs.

### Concentric cristae

Concentric cristae were limited to two related patients (patients 4 and 6) with the *m.8344A>G* mutation. Nevertheless, the existence of onion-like IMM organisation in human tissue extends previous observations in cultured human (HeLa) cells and yeast where down-regulation or knock down of mitochondrial contact site and cristae organising system (MICOS) components causes membrane stacking and/or onion-like concentric layering^33^,^43^,^44^,^45^. Abnormal dimerization of the ATP-synthase in yeast also promotes concentric cristae formation^46^.

Measurement of the intra- and inter- cristae space in normal mitochondria and those with concentric cristae demonstrated a significant reduction in size in concentric cristae indicating tighter packing of cristae membranes. The widths of intermembrane and intracristae spaces (Fig. 1) are regulated by specific proteins and are believed to be of functional relevance^19^,^47^. We did not observe cristae junctions in mitochondria with concentric cristae. Based on studies in model systems, potential causes of crista junction dysfunction and the resulting layering pattern include defects of nuclear-encoded MICOS components, or other crista junction or IMS proteins interacting with both OMM and IMM.

### Compartmentalization

An increase in the number of compartments observed within mitochondria was noted in two patients (patients 3 and 5). Sub-mitochondrial compartmentalization could be the product of partial or hemi-fusion subsequent to OMM fusion in the absence of IMM fusion, thus resulting in a single enlarged mitochondrion with multiple matrix spaces from the original smaller precursor mitochondria^48^. Consistent with this hypothesis, suppression of the IMM pro-fusion Opa1 homologue eat-3 in *Caenorhabditis elegans* causes a similar compartmentalisation phenotype^49^.

We observed localized distension of the OMM with apparent release of mitochondrial matrix content into the cytoplasm (Fig. 4G,H). This occurred without gross alteration of mitochondrial ultrastructure, distinguishing this process from permeability transition-induced swelling. Interestingly, recent evidence indicates that mitochondrial proteins (e.g. prohibitin, Hsp60) and mtDNA (in oxidized form) are released into the cytoplasm and systemic circulation^11^. Such ectopic mitochondrial material acts as damage associated mitochondrial proteins (DAMPs) or “alarmins”, triggering activation of inflammatory responses both intracellularly and systemically^11^. The factors determining mitochondrial compartmentalization and local release of mitochondrial material, and their relevance to myopathy, remain to be established.

### Nanotunneling

To our knowledge, this is the first report of nanotunnels in human skeletal muscle. The existence of “nanotunnels” was initially demonstrated in rat cardiomyocytes^50^. Consistent with earlier reports where similar structures were observed^51^,^52^, mitochondrial nanotunnels are stretches of both IMM and OMM devoid of cristae (Fig. 5A, inset), measure 50–200 nm in diameter^50^, able to transport large soluble proteins such as GFP between non-adjacent mitochondria^50^. Such communication system may be particularly relevant for physically constrained mitochondria in striated muscle, which cannot easily move and undergo fusion^50^.

*In vitro*, nanotunnels appear to grow between mitochondria in a kinesin KIF5B-dependent mechanism within seconds^53^. However, upon fusion with a distant mitochondrion newly established nanotunnels may increase in diameter, accommodate respiratory complexes via diffusion through the IMM, and thus contribute to the expansion of mitochondrial tubules and of the mitochondrial network^53^. Alternatively, nanotunnels could result from failed or stalled fission, a possibility supported by the fact that nanotunnels have a diameter at the lower limit of the constrictions (60–150 nm) induced by the dynamin Dnm1 during mitochondrial fission in yeast^54^. Failed fission could contribute to accelerate the spread of molecular defects across the mitochondrial network^55^.

Understanding the physiological and pathological conditions that promote or inhibit mitochondrial nanotunelling could offer new insights into the pathogenesis of mitochondriopathies, mutation load threshold, and progression of mitochondrial myopathy. It is worth noting that in this study nanotunnels were observed in specimens from three of seven patients, exclusively in those lacking PCIs. Nanotunnels were also observed in normal muscle without mitochondrial disease (n = 2), but not in those with the highest heteroplasmy levels and the most severe mitochondrial dysfunction. Therefore, nanotunnels may be indicative of normal or moderately impaired mitochondrial respiratory chain function.

### Hyperbranching and donut mitochondria

Excessive mitochondrial fusion or absence of fission leads to mitochondrial elongation and hyperbranching, possibly representing a compensatory acute response to mitochondrial stress^56^. Dose-response increase in the *m.3243A>G* mutation load *in vitro* also leads to progressive mitochondrial elongation^9^, and has been observed in aged mouse sarcopenic muscle^57^. Thus, hyperbranching and donut mitochondria may reflect cellular stress^58^ in mitochondrial myopathy patients with intermediate states of dysfunction, such as patient 7 (Fig. 6E,F).

Further to this we observe donut mitochondria in a further two patients (patients 2 and 6). The donut shape results from self-fusion or “splitting” of mitochondria and are induced experimentally by respiratory chain inhibition and oxidative stress *in vitro*^59^, and have been reported in brain presynaptic boutons of aged cognitively impaired non-human primates^60^. The presence of these features in human skeletal muscle may be reflective of the degree or nature of mitochondrial dysfunction, although more work is required to establish their physiological significance.

Some limitations of the present study should be noted. The aim of this investigation was to characterize the spectrum of mitochondrial ultrastructural features in human skeletal muscle. Accordingly, our approach consisted in capturing a large number of images depicting specific mitochondrial ultrastructural abnormalities, targeting myofibers with clear signs of pathology. While this sampling approach enabled the identification of multiple examples for known and novel ultrastructural defects, it is not compatible with a systematic unbiased quantification of the prevalence or frequency of the ultrastructural defects. Furthermore, muscle biopsies were collected from a convenience sample of consecutive individuals attending the diagnostic clinic, yielding a relatively small group of patients with different genetic defects. While this precludes conclusions regarding disease specificity, it has allowed to document and characterize a larger spectrum of ultrastructural abnormalities, and conclude that some structural defects are in fact not unique to specific mutations.

## Conclusion

Collectively, these results advance our understanding of mitochondrial dysfunction in several ways. First, in seeking to understand the relative contributions of sub-cellular compartmentalization in disease pathogenesis and progression, the recognition that certain features (i.e. PCIs) require the accumulation rather than the depletion of proteins may offer new insights into both the kinetics and nature of the underlying bioenergetics defects. Second, some of the reported features were previously observed *in vitro* or in model organisms following genetic disruption of specific mitochondrial proteins. For example, alteration of MICOS components disrupt CJs in various model systems (e.g., ref. 61), and could contribute to some observed features such as compartmentalization that completely lack CJs, and concentric cristae formation where CJs are rare. This opens the door to investigating the pathophysiological signficance of MICOS components in primary mtDNA diseases. Third, the origin of transcriptional dysregulation and pro-inflammatory gene activation in certain musculoskeletal disorders remains unclear. Given that mitochondria can transcriptionally regulate a large number of nuclear genes^9^,^10^ and trigger intracellular inflammatory processes^11^, localized membrane distension (Fig. 5G,H) or PCI-induced membrane rupture (Fig. 2C) could contribute to the cytoplasmic release of mitochondrial material that trigger these “non-energetic” pathogenic features. Finally, nanotunnel formation, donut-shaped mitochondria, and hyperbranching could represent evolutionary conserved compensatory responses to “mild” mitochondrial stress^56^,^62^.

Systematic assessment of mitochondrial morphology using quantitative EM methodologies sensitive mitochondrial size, shape, and branching complexity (e.g., refs 29,57), and particularly three-dimensional reconstruction methods such as serial block face (SBF-SEM) and focused-ion beam (FIB-SEM), will be required to ascertain the role of structural remodelling in certain mitochondrial and other musculoskeletal diseases. In addition, the functional significance of these changes will also need to be established through targeted molecular studies coupled to clinical investigations of mitochondrial ultrastructure. Expanding the spectrum of mitochondrial ultrastructure in human tissues should help us identify different mechanisms by which mitochondrial dysfunction contributes to disease.

## Material and Methods

### Literature review

Scopus, Embase and Medline were searched using the following search terms: “electron microscop* AND muscle AND human AND mitochondria*”. Filtering these for “full text” and “human” yielded 1549 results from Scopus, 1670 results from Embase and 922 results from Medline. Duplicates were removed, and search results screened on the basis of the following criteria: full text availability, English language, human, skeletal muscle, mitochondrial disease, any publication date up to the end of 2015. All included studies reported on patients with either a clinical or genetic diagnosis of mitochondrial disease; publications including cases where a diagnosis could not be reached have not been included. This resulted in 131 primary articles and four reviews (total n = 135). All articles were screened for the specific mitochondrial pathology reported, and for the diagnostic methodology used, which included restriction site mutation (RSM) assay, southern blotting, PCR, Sanger sequencing, restriction fragment length polymorphism (RFLP), solid phase mini sequencing, sequencing (general), next generation sequencing, or method not specified. Table S1 reports in chronological order the biopsied muscle, age of subjects, clinical features, genetic diagnosis, and a summary of mitochondrial ultrastructural and morphological feature(s).

### Patient cohort

This study was approved by the Newcastle and North Tyneside Local Research Ethics Committees (reference 2002/205) and prior informed consent was obtained from each participant. All experiments were carried out in accordance with the approved guidelines. Samples were collected in our diagnostic service over a period of approximately ten weeks where 11 consecutive cases underwent a muscle biopsy, seven of which were diagnosed with a primary mtDNA defect and reported upon here. For cases of single, large scale mtDNA deletions, sequencing was performed in two of the three cases to determine the breakpoint and size. Muscle biopsies were performed using the conchotome method under local anaesthesia (2% lidocaine) from tibialis anterior muscle of all diagnosed patients. Biopsy specimens were subsequently dissected and processed for electron microscopy. Demographic information and clinical data for each patient is summarized in Table 1.

### Transmission electron microscopy (TEM)

Muscle fiber bundles were teased from a fresh biopsy and fixed overnight in 2% glutaradehyde in 0.1 M Sorenson’s buffer (pH 7.4) at 4 °C as described previously^29^. Briefly, fibers were post fixed in 1% osmium tetroxide for 1 hour. Samples are dehydrated in a graded series of acetone (25%, 50%, 75%, 100%) before being embedded in epoxy resin (TAAB medium grade) and polymerised at 60 °C. Sections are cut on a Leica EM UC7 ultramicrotome, firstly semi-thin sections (0.5 um) are stained with toluidine blue for LM to identify the area of interest/confirm orientation of tissue. Ultrathin sections (70 nm) were then transferred to copper grids, and stained with uranyl acetate and lead citrate, and examined on a Phillips CM 100 Compustage (FEI) transmission electron microscope and digital micrographs were captured with an AMT CCD camera (Deben). On average seven individual fibers were thoroughly examined for each patient, for a total of 750 analysed images. Mitochondrial respiratory chain deficiency exists as a mosaic in skeletal muscle and cannot be conclusively ascertained for individual muscle fibers in ultrathin sections. We therefore focused our survey on myofibers with the highest mitochondrial content and/or showing signs of pathology at low magnification, such as vacuolization, mitochondrial hyperproliferation, or myofibrillar disarray. Regions of interest were scanned at 5,800x, images were captured at magnifications between 7,900x and 96,000x. Muscle fibers were examined in longitudinal and transverse orientations using methodology reported previously^29^.

### Serial block face scanning electron microscopy (SBF-SEM)

Tissue was fixed in 2% glutaraldehyde in 0.1 M cacodylate buffer (pH 7.4), and processed with heavy metal impregnation as described previously^63^. Briefly, tissue was immersed in 3% potassium ferrocyanide and 2% osmium tetroxide, followed by 0.1% thiocarbohydrazide, then 2% osmium tetroxide and finally left overnight in 1% uranyl acetate (with water washes between each step). The next day the samples were immersed in 0.6% lead aspartate solution and then dehydrated in graded acetone and embedded in epoxy tab 812 hard resin. After polymerisation blocks were trimmed and sectioned for standard TEM to identify areas of interest for SBF-SEM imaging. Fibers with high mitochondrial content were selected from cases with specific mitochondrial pathology and a series of serial images interspersed by 30–50 nm were captured at a magnification of 8,000–12,000x on a Zeiss Sigma scanning electron microscope with Gatan 3view system and digital micrograph software.

### Analysis and statistics

Image stacks were exported, processed, and used for reconstruction of abnormal features with IMOD 3dmod (IMOD 4.7, Boulder Laboratory for 3-D Electron Microscopy of Cells). Abnormal ultrastructure features detected in patients 4, 6 and 7 were reconstructed in 3dmod. Ultrastructural features were manually traced in consecutive image series and meshed. Surface transparency and smoothness were adjusted to facilitate visualization of features of interest in final reconstructed models. Each ultrastructural component was analysed separately to accurately quantify 3-dimensional features. Mitochondrial were pseudocolored (Keynote 6.6.1) to facilitate visualization of distinct organelles and structrures of interest.

Quantitative measurement of two-dimensional features (nanotunnel diameter and length, intracristae and intercristae distance, and intermembrane space) were performed in Image J (NIH, version 1.47v). For nanotunnel width measurements, the narrowest and widest regions of each nanotunnel were averaged, yielding a mean diameter for each nanotunnel (n = 26). Segments used for analysis were defined on the basis of lacking cristae and being of constant width. Data are reported as means ± S.D. Two-tailed T-test assuming unequal variance was used to evaluate differences in cristae measurements between normal and abnormal mitochondria, with the level of significance set at 0.05.

## Additional Information

**How to cite this article**: Vincent, A. E. *et al*. The Spectrum of Mitochondrial Ultrastructural Defects in Mitochondrial Myopathy. *Sci. Rep.* **6**, 30610; doi: 10.1038/srep30610 (2016).

## Supplementary Material

Supplementary Information

## Figures and Tables

**Table 1:** Case information for patients with mitochondrial disease.

Patient #	Sex	Age at biopsy	Genetic diagnosis	Heteroplasmy (if applicable)	% deficiency	% RRF	CK (IU/L)	Lactate (mmol/L)	Clinical phenotype	Diagnostic notes
1	F	47	Large-scale single mtDNA deletion	N/A	20	10	226	2.4	CPEO, proximal myopathy	—
2	F	62	Large-scale single mtDNA deletion	34	20	15	122–197	2.1–3.1	CPEO, proximal myopathy	—
3	F	70	Large-scale single mtDNA deletion	22	18	9	318–1238	1.4–2	CPEO, proximal myopathy	Asymmetric myopathy
4	F	22	*m.8344A>G*	97	97	10	145–291	7.4	Axial, proximal myopathy, lactic acidosis	—
5	F	50	*m.8344A>G*	63	22	7	43	1.6	Mild myopathy	Mother of patient 4
6	F	20	*m.8344A>G*	40	COX intermediate	0	267	1.2	Asymptomatic	Sister of patient 4
7	F	69	*m.3243A>G*	21	3	2	180–375	1.3	Diabetes mellitus, deafness, gut dysmotility	—

N/A: non-available; % COX: proportion of myofibers with cytochrome c oxidase deficiency; RRF: ragged red fibers; CK: creatine kinase; CPEO: chronic progressive external opthalmoplegia; HCM: hypertrophic cardiomyopathy; OA: optic atrophy; MERRF: myoclonic epilepsy with ragged red fibers. Deletions position and sizes for patient 1: N/A; patient 2: *m.8482-13460,* 4978 bp; patient 3: *m.8576-12968*, 4392 bp. Normal clinical range for CK: 25–200 IU/L; lactate: <2.2 mmol/L. All biopsies from the *tibialis anterior* muscle.

**Table 2:** Summary of mitochondrial ultrastructural abnormalities identified in patient skeletal muscle biopsies.

Patient #	Type I PCI	Type II PCI	Linearization and geometrical features	Concentric cristae	Compartmentalisation	Nanotunneling	Hyperbranching/hyperfusion	Projections
1	+++	−	−	−	−	−	++	−
2	−	−	−	−	−	+	+	−
3	−	−	−	−	+++	+	−	+++
4	−	+	+++	++	−	−	−	−
5	+	−	−	−	++	−	−	+
6	−	−	−	+	−	−	++	+
7	−	−	−	−	−	+++	+++	+++

PCI: Paracrystalline inclusion; +: Few; ++: Frequent; +++: Very frequent; −: none.
