# Supplementary material for: The Spectrum of Mitochondrial Ultrastructural Defects in Mitochondrial Myopathy
Source: Sci Rep. 2016 Aug 10;6:30610. doi: 10.1038/srep30610 (PMC4978969; doi:10.1038/srep30610)
Supplement: Supplementary Information [file srep30610-s1.pdf]

SUPPLEMENTARY INFORMATION

The Spectrum of Mitochondrial Ultrastructural Defects in Mitochondrial Myopathy

Amy E Vincent, Yi Shiau Ng, Kathryn White, Tracey Davey, Carmen Mannella, Gavin Falkous, Catherine Feeney, Andrew M Schaefer, Robert McFarland, Grainne S Gorman, Robert W Taylor, Douglass M Turnbull, Martin Picard

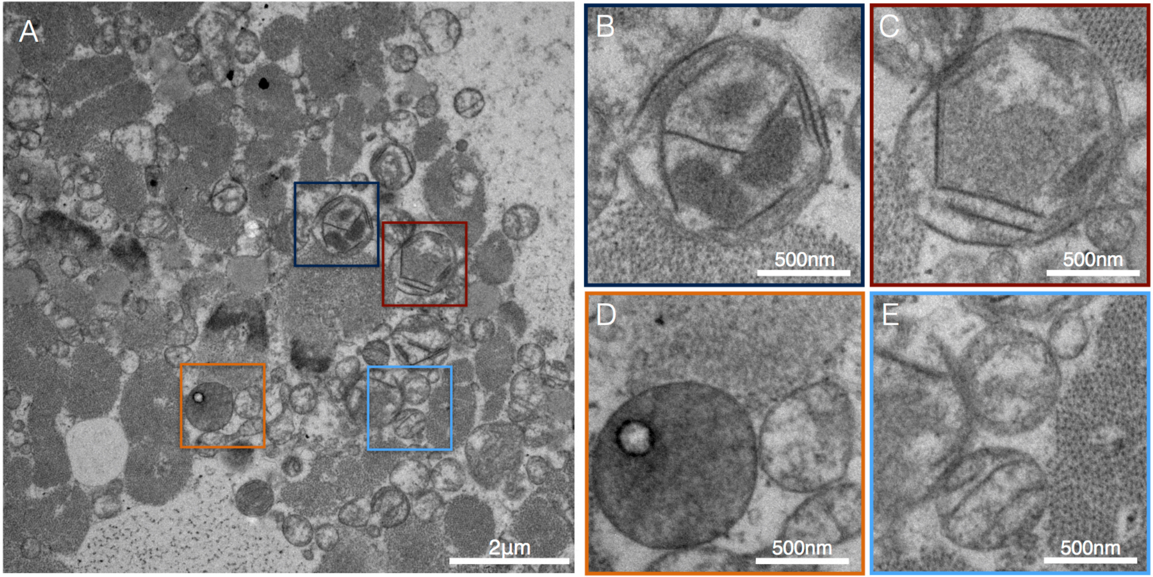

Figure S1. **Mitochondrial morphology and ultrastructure is heterogenous within muscle fibers. (A)** Low magnification electron micrograph of muscle fiber region containing mitochondria exhibiting different ultrastructural defects in patient 4 (m. 8344A>G). **(B-E)** Higher magnification images of selected areas in (A) showing heterogeneity in mitochondrial sizes, electron density, inclusion types, and cristae appearance and organization.

Supplementary Table 1: Results from a systematic review of literature of electron microscopy studies (n = 135) in skeletal muscle of patients with confirmed mitochondrial myopathy.

Author (Year)	Muscle	Age*	Clinical	Genetics	Ultrastructural abnormality
van Wijngaarden *et al.* (1967)	Deltoid and gastrocnemius	15 years	[Pediatric] Myopathy without muscle atrophy, deltoid muscle weakened, paresis of pelvic muscles (n=1).	N.S.	Matrix swelling and electron lucent mitochondria; clumped and filamentous, small osmiophilic dense bodies present in mitochondrial matrix; larger electron-dense round granules or irregularly shaped inclusions of moderately electron dense granular material.
Afifi *et al.* (1972)	N.S.	19 years	Hypermetabolic “mitochondrial” disease. Substantial muscle weakness (n=1).	N.S.	Osmiophilic inclusions of variable size and density within mitochondria; dense inclusions replacing most of the cristae, with cristae reduced to a thin circular rim at the periphery of affected mitochondria.
Hudgson *et al.* (1972)	Peroneus longus and biceps	35 years (proband) and 12 years (niece).	Familial “mitochondrial” myopathy. Facial and ocular, pectoralis major and bicep weakness, considerable lower limb weakness (n=2).	Dominant inheritance; high penetrance; maternal transmission. Mutation N.S.	Giant mitochondria measuring up to 3 µm containing concentric cristae and small electron-dense inclusions; mitochondria with “parking-lot” type inclusions between inner and outer mitochondrial membranes; mitochondrial with six layers with periodicity between both their transverse and axial cristae of approximately 75 A.
Adachi *et al.* (1973)	Ocular muscles	37 years	CPEO, cerebella ataxia and cardiomyopathy (n=1).	N.S.	Enlarged mitochondria with parallel or concentric cristae.
	Deltoid	37 years (same individual as above)	CPEO, cerebella ataxia and cardiomyopathy (n=1).	N.S.	Unusually large mitochondria ranging from 5-8 µm in greatest diameter with cristae usually located at the periphery of the organelle and rectangular inclusions; cross sections of the PCI structures disclosed well-organized arrays of tubules oriented in parallel rows with a diameter of 70-80 A, with a lumen of 30-40 A, and interspace between the tubules of 20-30 A, arranged in a helical pattern; abnormal mitochondria almost completely replaced by dense rectangular inclusions and occasionally showed complete transition to crystalline structures obscuring the original mitochondrial morphology.
Shibasaki *et al.* (1973)	Deltoid	60 years	Late onset myopathy; marked atrophy of shoulder girdle and proximal upper limb,	N.S.	Elongated, giant mitochondria up to 5 µm in greatest diameter, particularly in the SS region; PCIs including lattice pattern, four-fold array, or amorphous form.

			pelvic and proximal lower limb muscles; mild atrophy of distal lower limb muscles (n=1).		
Crosby and Chou (1974)	Left bicep	10 years	[Pediatric] Leigh disease (n=1).	N.S.	Enlarged mitochondria (in cross section); whirled cristae membranes; electron dense spheroid bodies; electron-lucent mitochondrial matrix spaces; complex, convoluted membrane systems; PCI within cristae membrane.
Black *et al.* (1975)	Anterior tibialis or Quadriceps	30 years	Mitochondrial disorder, RRFs, and ptosis (n=1).	N.S.	Enlarged mitochondria with "wavy" cristae and central electron dense granular material; round dense osmiophilic inclusions; occasional lipid inclusions and concentric cristae.
Karpati *et al.* (1975)	Quadriceps	11 years	[Pediatric] KSS biopsied before and after Carnitine supplementation (n=1).	N.S.	PCI; concentric cristae; swollen mitochondria.
McLeod *et al.* (1975)	Quadriceps (vastus lateralis)	27 years	Mitochondrial myopathy (n=1).	N.S.	PCI with parallel bands of amorphous material surrounded by a membrane; mitochondria with "empty" electron-lucent spaces; amorphous material between the mitochondrial membranes; mitochondria containing "empty space" surrounded by double membranes.
Bastiaensen *et al.* (1979)	Ocular and skeletal	>30 years [review]	PEO plus.	N.S.	[Review] Aggregates of swollen mitochondria with parallel or concentric cristae.
Linden and Ansink (1979)	Gastrocnemius	11 years	[Pediatric] KSS. Ptosis, mental and physical retardation, dysarthria (n=1).	N.S.	PCI; large mitochondria.
Fitszsimons and Tyer (1980)	Left deltoid	10 and 17 years	Mitochondrial myopathy. Myopathy, scoliosis, respiratory failure. (n=2)	N.S.	Rectangular inclusions, PCI, amorphous material and concentric cristae in mitochondria.
Fukuhara *et al.* (1980)	Quadriceps femoris	21 years	MERRF. Myoclonic jerk, seizures, hypotonia, ataxia (n=1).	N.S.	Electron-lucent homogenous matrix space; enlarged mitochondria; markedly proliferated IMM; PCIs.
Land *et al.* (1981)	Left deltoid	14 years	[Pediatric] Mitochondrial myopathy, opthalmoplegia (n=1).	N.S.	Mitochondria with "finger-print" like patterns (i.e., densely packed parallel cristae); concentric cristae.
Shah *et al.* (1982)	Quadriceps	N.S.	KSS, polymyositis,	N.S.	PCIs (30-45 nm long and 22-23 nm wide); round or oval swollen

			neurogenic atrophy, mitochondrial myopathy.		mitochondria; presence of various inclusions; densely packed or tubular cristae formation; concentric cristae or "lamellar bodies"; parallel destruction of cristae and presence of amorphous material.
Holliday *et al.* (1983)	N.S.	18 and 26 years	Mitochondrial myopathy, encephalopathy (n=2).	N.S.	Rectangular or curvilinear PCIs composed of four parallel lamina within a boundary membrane.
Mitsumoto *et al.* (1983)	Deltoid	11 to 49 years (m=34.5)	CPEO with RRF (n=8).	N.S.	Mitochondria with absent cristae; irregular vacuoles; PCIs; concentric cristae; dark inclusion; whirl formation.
Muller-Hocker *et al.* (1983)	N.S.	N.S.	PEO (n=8).	N.S.	Enlarged mitochondria with high cristae content; concentric cristae and PCIs.
Prick *et al.* (1983)	N.S.	2 and 5 years	[Pediatric] Alper's disease (n=2).	N.S.	Aggregates of enlarged mitochondria with PCIs in SS region.
Sarnat *et al.* (1983)	Quadriceps femoris, deltoid, extensor digitorum longus of the forearm (post mortem)	3 weeks and 4 months	[Pediatric] Mitochondrial myopathy, cerebro-hepato renal (Zellweger) syndrome (n=2).	N.S.	Electron dense granules in mitochondrial matrix; myelin figures, degeneration and other degenerative changes (e.g. membrane rupture) to mitochondria.
Sengers *et al.* (1983)	Quadriceps	4 years	[Pediatric] Mitochondrial myopathy, carnitine deficiency (n=1).	N.S.	Large mitochondria with densely packed or whirled cristae; granular or fibrillary electron dense material in intercristal space.
Hayes *et al.* (1984)	Intercostal muscle	20 years	Mitochondrial myopathy, bilateral ptosis, muscle bulk normal, mild diffuse weakness (n=1).	N.S.	SS mitochondria with concentric cristae and inclusions; central droplets of neutral or osmiophilic lipid or areas of central necrosis.
Kennaway *et al.* (1984)	N.S.	17 years	Mitochondrial myopathy, lactic acidosis (n=1).	N.S.	Abnormally large mitochondria some with PCIs.
Bresolin *et al.* (1985)	Quadriceps	2, 3, 6 and 7 months	[Pediatric] Mitochondrial myopathy, hypertonia and severe limb weakness, tendon and pharyngeal weakness absent (n=1).	N.S.	Large mitochondria with irregular or concentric tubular cristae.
Muller-Hocker *et al.* (1985)	N.S.	12 years	[Pediatric] Mitochondrial myopathy, excercise	N.S.	Swollen mitochondria with PCIs.

			induced muscle weakness and pain, tachycardia and dyspnoea (n=1).		
Niebrój-Doblosz *et al.* (1985)	Biceps brachii and quadriceps	7 years	[Pediatric] KSS. Biceps and quadriceps weakness, mental retardation, progressive limb weakness (n=1).	N.S.	Giant mitochondria; PCIs; concentric cristae.
Fischer *et al.* (1986)	Quadriceps	17 years	Mitochondrial enchephalomyopathy, seizures, myoclonic contractures, retarded motor and speech development (n=1).	N.S.	Small mitochondria with swollen matrix; concentric cristae or "concentric laminated bodies".
Lehmann *et al.* (1986)	Deltoid	44 years	Myopathy with rimmed vacuoles, mild shoulder girdle weakness, exercise intolerance (n=1).	N.S.	PCIs of Type I and II.
Stadholders and Sengers (1987)	N.S.	N.S.	Mitochondrial myopathy (KSS, MELAS, MERRF)	N.S.	[Review] PCIs and concentric cristae; suggests model of PCI formation; classify 2 types of PCI (Type I, Type II); suggests different types of PCI for different fibre types.
Van Ekeren *et al.* (1987)	N.S.	6 days	Hereditary mitochondrial hypertrophic cardiomyopathy (n=1).	N.S.	Mitochondria with vesicular inclusions; matrix crystals.
Columbo *et al.* (1988)	Left quadriceps	27 years	Mitochondrial occuloskeletal myopathy (n=1).	N.S.	PCIs; globular dense bodies; cristae running concentrically.
Dias-Tosta (1988)	N.S.	N.S.	CPEO. Sporadic muscle weakness (n=10), familial isolated myopathy (n=9), myopathy with retinopathy, cerebellar ataxia, or peripheral neuropathy (n=15).	N.S.	PCIs; concentric cristae; "parking lot" and "intramural type" inclusions.
Federico *et al.*	Quadriceps	N.S.	LHON (n=3).	N.S.	Enlarged mitochondria with PCIs.

(1988)					
Mizusawa *et al.* (1988)	Rectus femoris or tibialis	46 and 48 years	Familial mitochondrial myopathy, pptic atrophy, muscle wasting, weakness (n=2).	N.S.	Aggregates of mitochondria with PCIs; electron dense globular bodies; concentric cristae.
Kim *et al.* (1989)	Left deltoid	53 years	CPEO. Mild proximal limb weakness (n=1)	N.S.	PCIs; irregularly orientated cristae.
Paulus *et al.* (1989)	Left quadriceps	33 years	Mitochondrial encephalomyopathy with perivacuolar inclusions, bilateral hearing loss, seizures, cerebral atrophy, moderate muscle atrophy (n=1).	N.S.	Small osmiophilic rod-like inclusions; piles within round or oval vacuoles (perivacuolar inclusions).
Rivner *et al.* (1989)	N.S	25 years	KSS. Cerebellar and cortical atrophy, mild ataxia and generalised weakness of proximal and distal muscles (n=1).	N.S.	Deformed internal architecture; PCI arrays; concentric cristae or "cristae arranged circumferentially".
Suzuki *et al.* (1989)	Quadriceps	35 years	Mitochondrial encephalomyopathy, general muscle atrophy, psychiatric disturbance (n=1).	N.S.	Enlarged mitochondria with PCIs.
Arruda *et al.* (1990)	Quadriceps	32 and 34 years	Mitochondrial myopathy with myoclonic epilepsy. Myoclonic jerks and seizures (n=2).	N.S.	Enlarged mitochondria.
Haginoya *et al.* (1990)	Quadriceps (vastus femoris)	8-43 years (m=25)	MERRF and MELAS. Mixed, myoclonic seizure, ataxia, stroke-like episodes, ptosis (n=3).	N.S.	Aggregated mitochondria in areas of segmental COX deficiency.
Korenke *et al*. (1990)	N.S.	2 weeks - 9 years (m=2 years)	[Pediatric] Mitochondrial myopathy. Mixture; respiratory insufficiency, hypotonia, motor	N.S.	Abnormally small and large mitochondria; PCIs; polymorphy; altered cristae.

			retardation and feeding problems (n=8).		
Schapira *et al.* (1990)	Quadriceps (vastus lateralis)	14 years	Mitochondrial myopathy. Weakness of limbs and trunk, high CK.	Defect in protein transport? Molecular cause N.S.	Disordered “labyrinth” cristae with electron dense and granular inclusions.
Sumegi *et al.* (1990)	N.S.	45 days	[Pediatric] COX deficiency.	N.S.	Swollen mitochondria, and often “tennis-racket” like mitochondria (swollen at one end); mitochondria with honeycomb cristae.
Takeda *et al.* (1990)	Extraocular muscles	14 to 30 years (m=20)	MELAS and MERRF. Mixed symptoms (n=3).	N.S.	Mitochondria of irregular shape with tubule-vesicular cristae; morphological changes more prominent in MELAS than MERRF. No PCI.
Kim *et al.* (1991)	N.S.	16 and 22 years	Mitochondrial myopathy. Severe respiratory dysfunction, mild myopathy.	N.S.	Sickle shaped mitochondria containing curvilinear PCI, regular PCIs.
McKelvie *et al.* (1991)	Quadriceps (vastus lateralis)	48 years (n=1)	KSS. Opthalmoplegia, limb weakness, ataxia (n=1).	3.5Kb single mtDNA deletion[i]	PCIs in subsarcolemmal aggregates of mitochondria.
Letellier *et al.* (1992)	Deltoid	1 week to 4 months (m=2 months)	[Pediatric] Mitochondrial myopathy. Mixture of symptoms (n=6).	N.S.	Enlarged and swollen mitochondria.
Linda *et al.* (1992)	N.S.	N.S.	Mitochondrial myopathy. MELAS (n=3), Marinesco-Sjogren syndrome (n=7) and KSS (n=2).	mtDNA mutations N.S.	PCIs; pleoconial mitochondria; enlarged “megamitochondria”; thickening, unfolding, and parallelization of cristae; concentric cristae or “concentric lamellar cristae”; augmented matrix space; amorphous inclusion bodies.
Modi *et al.* (1992)	Biceps brachii	20 years	Mitochondrial myopathy. Proximal myopathy, cardiomyopathy, bilateral deafness and ptosis.	N.S.	Laminated PCIs.
Telerman-Toppet *et al.* (1992)	N.S.	8 weeks	[Pediatric] COX deficiency and mtDNA depletion (n=1).	N.S.	PCIs; abnormal spatial arrangement of mitochondrial cristae.
Angelini *et al.*	Biceps and	19 and 25	Hypertrophic	Dystrophin	Enlarged mitochondria with irregular cristae.

(1993)	quadriceps	years	cardiomyopathy with mitochondrial myopathy. Weakness in upper limb girdle muscles and Gower's signs and waddling gait, no cerebellar abnormalities (n=2).	and mtDNA deletions ruled out. Cause N.S.[i]	
Coquet *et al.* (1993)	N.S.	15 and 17 years	MERRF. Myoclonic jerks, epileptic seizures, ataxia, deafness, lower limb weakness (n=2).	*m.8344A>G*[a] $tRNA^{Lys}$	PCIs; concentric cristae.
Reichmann *et al.* (1993)	Biceps brachii	53 years	KSS. Mild generalized muscle weakness and exercise intolerance, diabetes, slowly progressive ataxia (n=1).	Multiple deletions[b] (causative mutation N.S.)	PCIs; concentric cristae.
Ionasescu *et al.* (1994)	Quadriceps (vastus lateralis)	9 years	[Pediatric] MERRF. Progressive proximal weakness (n=1).	*m.15990A>G*[a] $tRNA^{Pro}$	PCIs.
Norby *et al.* (1994)	Quadriceps (vastus medialis)	15 years	[Pediatric] KSS.<br>Epileptic seizures, ataxia, myopathy (n=1).	11Kb single mtDNA deletion[b]	Enlarged mitochondria with PCIs.
Stadhouders *et al.* (1994)	N.S.	N.S.	CPEO, KKS, MELAS	N.S.	PCIs (Types I and II).
Gilbert and Ems (1996)	N.S.	21 months	Pearson's syndrome. Pancreatic atrophy, enlarged kidneys, metabolic acidosis, high blood lactate and hypomagnesemia (n=1).	N.S.	Aggregated SS and IMF mitochondria; concentric cristae, enlarged mitochondria with disrupted membranes; central follicular (granular) matrix.
Melberg *et al.* (1996)	Quadriceps (vastus lateralis) or bicularis oculi	16 to 63 years (m=52 years)	Dominant PEO. Ocular myopathy, hypogonadism (n=14).	Dominant genetic cause N.S.	Densely packed cristae; size variation; longitudinally or circular cristae; crystalloid-like alterations.

Toscano *et al.* (1996)	N.S.	73 to 75 years (m=74 years)	Late-onset mitochondrial neuropathy. Muscle weakness and cramps, ataxia, exercise intolerance (n=3).	No mtDNA mutations found[b,c]. Genetic cause N.S.	PCIs, enlarged or bizarrely shaped mitochondria.
van Domburg *et al.* (1996)	Soleus, quadriceps, ocular rectus superior	25 to 36 years (m=30 years)	CPEO plus. Ocular myopathy, encephalomyopathy, sensory neuropathy, ataxia, polyneuropathy (n=6).	No evidence of maternal inheritance, common point mutations and deletions ruled out[i]. Genetic cause N.S.	PCIs, mitochondria with deranged cristae; concentric cristae.
Wada *et al.* (1996)	Quadriceps femoris	11 weeks	[Pediatric] Mitochondrial myopathy. Muscular hyoptonia, profound acidosis, difficulty suckling, no muscle weakness (n=1).	N.S.	Enlarged mitochondria with sparse cristae; concentric cristae.
Heimann-Patterson *et al.* (1997)	N.S.	4 to 63 years (m=28 years)	Family with mitochondrial myopathy. Exercise intolerance, proximal lower limb weakness in first decade of life (n=11).	mtDNA deletions and common point mutations ruled out, a number of non-pathogenic point mutations found[d]. Genetic cause N.S.	Uneven and electron dense cristae; globular inclusions.
Kim and Chi (1997)	Deltoid	22 and 23 years	KSS. Neck and proximal limb weakness, severe ptosis, AV block.	Single mtDNA deletion[1,c]	PCIs, enlarged mitochondria with distorted cristae.
Smith *et al.* (1997)	Quadriceps	N.S.	Mother of MELAS patient, with insulin dependent	*m.3243A>G*[a] $tRNA^{Leu}$	Pleomorphic mitochondria; concentric cristae; electron-dense bodies.

			diabetes (n=1).		
Takahashi *et al.* (1998)	Quadriceps femoris	9 months	[Pediatric] Leigh disease. Generalized hypotonia at birth, apnea attacks, irregular breathing, poor head control (n=1).	*m.8993T>G*[a] MT-ATP6	Variably sized mitochondria; aberrant cristae.
Castro-Gago *et al.* (1999)	Deltoid	Fetus	Hydranencephalic-hydrocephalic syndrome in association with mitochondrial encephalomyopathy.	Common mtDNA point mutations and deletions ruled out[i]. Genetic cause N.S.	Enlarged SS mitochondria; abnormal cristae (honey-comb-like).
Kyriacou *et al.* (1999)	N.S.	6 days to 10 years (m=2 years)	[Pediatric] Mitochondrial enchephalomyopathy. Hypotonia and psychomotor retardation (n=5), multisystemic (n=2), Pearson syndrome (n=1), Leigh disease (n=1).<br>(Adult) Myalgia and proximal limb weakness (n=8), PEO (n=3).	N.S.	Pleomorphic mitochondria; simplified cristae; vesicular cristae.
Melegh *et al.* (1999)	N.S.	5 months	Mitochondrial enchephalomyopathy. Myoclonic jerk, uncoordinated eye movement (n=1).	Multiple mtDNA deletions[b]. Nuclear mutation N.S.	Enlarged mitochondria with no or few cristae.
Carta *et al.* (2000)	Quadriceps (rectus medialis)	48 years	CPEO (n=1)	Single common mtDNA deletion[1,b]	PCIs; “onion-like” concentric cristae, “ghost-like” mitochondria (devoid of cristae).
Makino *et al.* (2000)	N.S.	N.S.	Leigh syndrome. Hypotonia, necrotic lesions in basal ganglia and brainstem (n=1).	*m.8993T>G*[a] MT-ATP6	Enlarged mitochondria.

Wang *et al.* (2000)	N.S.	9 years	No prominent muscle weakness or hypotonia, but wobbling gait early on. Proximal limb myopathy 1 month prior to biopsy (n=1).	N.S.	Loss of matrix and cristae.
De Kremer *et al.* (2001)	N.S	N.S.	[Pediatric] Barth-syndrome like disorder, marked muscle weakness.	*m.3243A>G*[d] $tRNA^{Leu}$	Round swollen mitochondria; fine fibrillary matrix; poorly define cristae.
Higashikata *et al.* (2001)	Biceps brachii	80 years	MELAS. Cardiomyopathy.	*m.3243A>G*[a] $tRNA^{Leu}$	Aggregates of enlarged mitochondria with PCIs.
Rollins *et al.* (2001)	N.S.	1 to 70 years (m=12.5 years)	Mitochondrial cytopathy (n=113)	Mutation of "maternal inheritance" N.S.	PCI; enlarged mitochondria.
Vogel (2001)	N.S.	N.S.	Mitochondrial myopathy	N.S.	[Review] Swollen, elongated, devoid of cristae, PCIs.
Fagiolari *et al.* (2002)	N.S.	27 to 73 years (m=55 years)	CPEO (n=7), PEO and ptosis (n=7), myopathy (n=1).	Multiple mtDNA deletions[b] due to *ANT1* mutation – *p.114A>P* and *p.98L>P*[i]	Mitochondrial proliferation and PCIs.
Fillano *et al.* (2002)	N.S.	2 to 12 years (m=7 years)	HEADD syndrome (hypotonia, epilepsy, autism, and developmental delay) (n=3)	Multiple mtDNA deletions[c] (nuclear cause N.S.) or 7.4Kb single mtDNA deletion[c]	Stacked cristae; electron dense deposits.
Kim *et al.* (2002)	Biceps brachii	18 Years	MERRF. Myoclonic epilepsy, myopathy, ataxia, dysarthria (n=1).	*m.8344A>G*[e] tRNALys	PCI; concentric cristae.
Marín-García *et al.* (2002)	N.S.	15 years	PEO (n=1)	Multiple mtDNA	Vacuolated cristae; variation in size and morphology.

				deletions[b]	
Nevo *et al.* (2002)	Quadriceps	11 to 26 months (m=16 months)	[Pediatric] Mitochondrial myopathy. Motor deterioration, dyspneic, muscle weakness, bilateral ptosis.	*TK2* mutation causing mtDNA depletion[i]	Pleomorphic mitochondria; enlarged mitochondria; concentric cristae or “concentric lamellae”.
Kyriakides *et al.* (2003)	N.S.	6 days to 18 months (m=6 months, n=19), 19 to 75 years (m=42 years, n=14)	Mitochondrial encephalomyopathies: myopathic, multisystemic or CPEO. Pediatric (n=19) and adult (n=14).	N.S.	Mitochondrial dysmorphology more common in children than adults and vice versa.
Uusimaa *et al.* (2003)	N.S.	10 months	Alpers-Huttenlocher-like disease (n=1)	*m.7706G>A*[d] MT-COII	Enlarged mitochondria; variable shape and size.
Mierau *et al.* (2004)	Quadriceps	8 years	[Pediatric] Lactic acidosis, proximal weakness, cognitive delay.	N.S.	PCIs; enlarged or abnormally small; concentric cristae; parking lot type inclusions; spherical electron dense inclusions.
Patterson *et al.* (2004)	N.S.	16 years	KSS.	5Kb mtDNA deletion[f]	PCIs.
Carta *et al.* (2005)	Quadriceps (rectus medialis)	42 to 75 years (m=63 years)	CPEO and LHON (n=3)	Single common mtDNA deletion[1,i]	“Ghost” mitochondrial profiles (lack of cristae) with partial to complete matrix emptying rearrangement of the cristae with an “onion ring-like” appearance
Coulbault *et al.* (2005)	N.S.	10 months	[Pediatric] Psychomotor retardation, failure to thrive, hypotonia, hepatocellular dysfunction, thrombocytopenia.	*m.5693T>C*[e], $tRNA^{ASN}$	PCIs; stacked cristae.

			Multiple organ failure (lethal) (n=1).		
Dinopoulos *et al.* (2005)	Quadriceps	1 to 15 months (n=9 months)	[Pediatric] Hypotonia, myoclonic epilepsy, cardiomyopathy, psychomotor regression, failure to thrive, lactic acidosis (mixed) (n=4).	*NDUFS2 c.237T>C*, *NDUFS8 c.236C>T* and *c.302G>A*, *NDUFVI c.175C>X* and *c.1268C>T* mutations (CI)[i]	Concentric cristae or "concentric laminated bodies"; osmiophilic mitochondria.
Enns *et al.* (2005)	Quadriceps (vastus lateralis) and deltoid	6 weeks to 5 years (m=1.5 years)	Electron transport chain abnormalities, RRFs. (n=7)	N.S.	Enlarged mitochondria; swelling with electron-lucent matrix space; proliferation of mitochondria; elongated and branched.
Güçer *et al.* (2005)	N.S.	4 months and 9 years	[Pediatric] Seizures, ptosis, failure to thrive, high serum lactate and pyruvate, RRF (n=2).	Single common mtDNA deletion[1,b]	Increased matrix density and cristae; SS mitochondrial accumulation.
Karppa *et al.* (2005)	Quadriceps (vastus lateralis) or tibialis anterior	16 to 70 years (m=45 years)	MELAS	*m.3243A>G*[i] $tRNA^{Leu}$	PCIs (Types I and II); variation in size and ultrastructure.
Kyriacou *et al.* (2005)	N.S.	6 days to 18 years (median= 4 years, n=31) and 19 to 75 years (median= 46 years, n=17)	Mitochondrial encephalomyopathy (n=48).	N.S.	Elongated and enlarged mitochondria more common in children than adults; subsarcolemmal aggregation of mitochondrial (RRF) more common in adults.
Pulkes *et al.* (2005)	N.S.	16 and 23 years	MERRF. Proximal upper and lower limb weakness, ataxia, seizures and myoclonus (n=2).	*m.7472_insC*[i] $tRNA^{Ser}$	Type 2 fibres larger and dense matrix components and contain either or both type 1 or 2 crystalline intra-membranous inclusions. Type 1 myofiber mitochondria, have many large and abnormal forms, show a more orderly arrangement of their cristae, a lesser

					quantity of less dense matrix, and only type 1 intra-membranous crystalline inclusions.
Stenqvist *et al.* (2005)	Quadriceps (vastus lateralis)	10 years	[Pediatric] MELAS. Mild central and cortical brain atrophy, encephalopathy, no mention of myopathy (n=1).	*m.3271T>C*[f] $tRNA^{Leu}$	Dense granular inclusions, parallel cristae arrays, open matrix spaces.
Zeharia *et al.* (2005)	N.S.	19 years	Mitochondrial myopathy (n=1).	Autosomal recessive *PUS1*[d]	PCIs.
Cardaioli *et al.* (2006)	Quadriceps	31 years	Deafness, mental retardation, brain atrophy (n=1).	*m.7472insC*[e] $tRNA^{ser\ (UCN)}$ and *m.7472A>C*[e] $tRNA^{ser\ (UCN)}$	Swollen mitochondria with reduced cristae and PCIs.
Deschauer *et al.* (2006)	N.S.	66 years	Exercise intolerance, walking difficulty, muscle cramps, hearing impairment, mild myopathy and peripheral neuropathy (n=1).	*m.622G>A*[d] $tRNA^{Phe}$	Enlarged mitochondria with PCIs.
Miles *et al.* (2006)	N.S.	2 weeks to 21 years (median= 2.7 years)	KSS (n=4), Leigh syndrome (n=2), lethal infantile COX deficiency (n=2), MERRF (n=1), encephalopathy with (n=72) and without myopathy (n=46).	N.S.	PCIs; abnormal patterns of cristae; concentric cristae or "circular cristae" and fingerprint cristae; abnormal matrix density.
Saneto and Boulding (2006)	N.S.	11 years	[Pediatric] Muscle weakness, hypercarbia, lactic acidosis, absent deep tendon reflexes, respiratory difficulty (n=1).	*m.3243A>G*[i] $tRNA^{Leu}$	Enlarged rounded mitochondria; distorted cristae; electron dense inclusions.
Thajeb *et al.*(2006)	N.S.	65 years	Oculopharangyl somatic myopathy, bilateral ptosis and difficulty swallowing	Single mtDNA deletion[c,d] and homoplasmic *m.5814T>C*[d]	"Parking lot" type PCIs.

			(n=1).	$tRNA^{Cys}$	
Wabbels *et al.* (2007)	M. levator palpebrae, and/or the M. orbicularis oculi	4 to 81 years (m=40 years)	Adult/child Ptosis/CPEO (n=21).	N.S.	PCIs; concentric and wavy cristae; pleomorphy; intermitochondrial granules.
Perry *et al.* (2008)	Quadriceps	3 days	[Pediatric] Sengers syndrome. Bilateral ptosis, hypotonia, trace reflexes, skeletal muscle myopathy and cardiomyopathy (n=1).	N.S.	Diffuse dysmorphic mitochondria.
Pronicki *et al.* (2008)	Quadriceps (vastus lateralis)	9 months to 12 years (m=3.5 years)	[Pediatric)] Leigh Syndrome. Motor regression, bulbar symptoms, difficulty walking, hypotonia, failure to thrive, floppiness, disturbed eye movement (n=21).	*SURF1*[i] mutation	Enlarged or elongated mitochondria; dark matrix with densely packed, concentric lamellae cristae; mitochondrial matrix displaced by amorphous granular material; small electron-dense osmiophilic granules.
Yerdelen *et al.* (2008)	Biceps brachii	38 years	KSS. Delayed motor and mental development, arrhythmia (n=1).	N.S.	PCIs; swollen mitochondria with few electron dense cristae; osmiophilic and electron-dense structures.
Abu-Amero *et al.* (2009)	N.S.	34 years	MELAS (n=1)	No mtDNA mutations or POLG mutations. Genetic cause N.S.	Mitochondria with parallel cristae and PCIs, osmiophilic inclusions and mitochondrial vacuoles.
DeBrosse *et al.* (2009)	Deltoid	67 years	CPEO. Ptosis, mild upper limb weakness, mild memory difficulty and imbalance (n=1).	N.S.	PCIs; dystrophic mitochondria.
Yau *et al.* (2009)	Quadriceps	14 years	[Pediatric] KSS, short stature, mental deficiency, lower limb	Single 7.2Kb mtDNA deletion[c]	PCIs; irregular structure and cristae; central core-like fibres (IMF mitochondrial depletion).

			weakness, mild ataxic gait (n=1).		
Ali *et al.* (2010)	Triceps brachii	62 years	CPEO. Diplopia, no muscle weakness (n=1).	Single mtDNA deletion[1,b,c,d]	PCIs (Type I); swollen rounded mitochondria.
Ascaso *et al.* (2010)	N.S.	48 years	KSS. Bilateral ptosis, proximal muscle weakness and RRFs (n=1).	Single mtDNA deletion[1b,c,d]	Large atypical mitochondria; round electron dense bodies.
Cardenas and Amato (2010)	N.S.	14 years	[Pediatric] Alpers disease. Neuropathalogical degeneration of cerebral grey matter (n=1).	*POLG* mutations (compound heterozygous) *c.911T>G* (p. L304R), *c.1174C>G* (p.L392V) and a duplication (pR1081dup)[f]	Reduced mitochondrial content, absence of cristae, vacuolization.
Chi *et al.* (2010)	Quadriceps	1 month to 15 years (median= 15 months, n=69)	[Pediatric] Leigh disease (n=6).	*m.8993T>G* (n=3) MT-ATP6*, m.8993T>C* MT-ATP6 (n=1)*, m.10191T>C* MT-ND3 (n=1) and *m.8344A>G* (n=1)[c,d,e]	Tubular cristae; swollen mitochondrial accumulation; variable shapes; concentric “whirl” cristae; inclusion bodies.
			[Pediatric] MELAS (n=4).	*m.3243A>G* tRNA^{Leu} (n=4)[c,d,e]	
			[Pediatric] Pearson syndrome (n=1)	6 Kb mtDNA deletion (n=1)[c,d,e]	

			Mixed mitochondrial disease (n=58)	Genetic cause N.S.	
Han *et al.* (2010)	Rectus femoris	4 to 48 years (m=N.S)	Spinocerebellar ataxia type 7. Unsteady gait, visual problems, cerebellum and brain stem atrophy (n=34).	*SCA7* mutation[c] CAG repeat	Mitochondrial with tubular or absent cristae.
Herrero-Marti *et al*. (2010)	N.S.	50 years	MELAS MERRF overlap (n=1)	*m.5521G>A*[g] $tRNA^{Trp}$	PCIs.
Lang *et al.* (2010)	Ocular medial rectus	47 and 67 years	CPEO, ptosis, exercise intolerance and mild myopathy (n=2).	Multiple mtDNA deletions[i] (nuclear mutation not defined)	Enlarged mitochondria; hypertrophic concentric lamellar cristae.
Baskin *et al.* (2011)	N.S.	9 years	Mitochondrial myopathy. Myopathic findings by electromyography, deafness, ocular and neurological findings (n=1).	*NPHS3*[g] mutation	Abnormal mitochondrial shape and cristae patterns.
Cenacchi *et al.* (2011)	Quadriceps, Tricep, deltoid	19 to 79 years (m=56 years)	Mitochondrial myopathy (=14).	N.S.	Accumulation of SS mitochondria; rounded, elongated, or cup-shaped mitochondria; abnormally large or "mega-mitochondria"; concentric cristae; lipid-dense; PCIs in matrix.
Conway *et al.* (2011)	gastrocnemius	29 years	MELAS. Seizures, hearing loss (n=1).	*m.3243A>G*[i] $tRNA^{Leu}$	Abnormally arranged cristae and aberrant electron density.
Shaaf *et al.* (2011)	N.S.	3 and 8 years	Optic nerve atrophy, hypotonic, ataxia (n=2).	*OPA1* mutation *c.2708_2711delTTAG* (p.V903GfsX3) and *c.1146A>G* (p.I382M)[d]	Mitochondrial with dense osmiophilic bodies.

Blakey *et al.* (2012)	Quadriceps	21 years	Neuropathy and leukoencephalopathy, walking difficulties, distal numbness, muscle wasting of hands and distal lower limbs (n=1).	*MPV17* mutation[d] causing 12.5Kb mtDNA deletion[c]	Enlarged, "parking lot" type inclusions.
Bostan *et al.* (2012)	Deltoid	52 years	Ptosis, ataxia, neuropathy, gastroparesis, myopathy (n=1).	*POLG* mutation[i]	PCIs.
Gotz *et al.* (2012)	N.S.	5 days	[Pediatric] Fatal neonatal lactic acidosis (n=1).	*m.7453G>A*[d] $tRNA^{Ser}$	Enlarged and swollen mitochondria, concentric cristae.
Kendall *et al.* (2012)	Multiple	N.S.	Multiple mitochondrial disorders.	N.S.	[Review] Previous reports suggest electron microscopy is not a good diagnostic tool. However other sources report the frequency of 30-44% of aberrant mitochondrial morphology in pediatric mitochondrial disease and conclude its usefulness.
Pfeffer *et al.* (2012)	Levator palpebrae	34 to 72 years (m=52 years)	CPEO (n=8)	*POLG1* (n=1), single mtDNA deletion (n=4), multiple mtDNA deletion (n=1, NS (n=2)[b,c,e]	Large mitochondrial aggregates, pleomorphism, various inclusions.
Roefs *et al.* (2012)	Orbicularis	46 to 64 years (m=58 years)	Progressive bilateral ptosis and generalised muscle weakness (n=3).	Single mtDNA deletion[b,c]	Enlarged mitochondria; densley packed parallel cristae; electron dense inclusions.
Baric *et al.* (2013)	Deltoid	17 years	Elevated CK, mild myopathy in brachial muscles.	*m.5522G>A*[c,g] $tRNA^{Trp}$	Swollen globular looking mitochondria, occasional branching and angular OMM.
Lopez *et al.* (2013)	Tibialis anterior	22 years	[Pediatric] Fatal infantile mitochondrial DNA depletion (n=1).	*TK2* mutation[c,g]	Elongated mitochondria with PCIs.
Lu and Huang (2013)	Biceps brachii	1 to 13 years (m=8	MELAS (n=6)	*m.3243A>G*[i] $tRNA^{Leu}$	Globualar looking mitochondrial shape elongated with swollen regions and PCIs.

		years)			
Nolte *et al.* (2013)	N.S.	16 and 17 years	Seizures, myoclonic jerk, PEO (n=2).	*POLG1 c.2243G>C* (p.W748S) and *c.1879C>T* (p.R627W)[g]	Elongated mitochondria with "globoid" inclusions (amorphous lipid-like); SS mitochondrial accumulation or "aggregates".
Polimeno *et al.* (2013)	N.S.	N.S.	Mitochondrial myopathy (other symptoms N.S.).	N.S.	PCIs; mitochondria assuming bizarre and/or giant forms.
Siriwardena *et al.* (2013)	N.S.	15 months	Sengers syndrome (n=1)	*AGK* mutation[g]	Pentagonal mitochondrial crystals with a dense outer layer.
Yuan *et al.* (2013)	N.S.	51 and 54 years	RRF, rimmed vacuoles, muscle weakness in facial, cervical and proximal muscles, wasting of shoulder girdle (n=2).	*m.8344A>G*[d] $tRNA^{Lys}$	PCIs; Mitochondrial proliferation, concentric cristae.
Zhao *et al.* (2013)	Biceps brachii	66 years	CPEO, RRF and diffuse muscle weakness (n=1)[g].	Large 4Kb mtDNA mutation	PCIs.
Chen *et al.* (2014)	N.S.	44 years	CPEO, ptosis, diplopia, bulbar paresis, inflammatory myopathy, neck muscle weakness (n=1).	Single mtDNA deletion[1,h]	PCIs preferentially in SS mitochondria.
Hopmann *et al.* (2014)	N.S.	72 years	Mitochondrial myopathy, muscle atrophy and proximal weakness of arms trunk and legs (n=1).	Multiple mtDNA deletions[b,c]	"Parking lot" inclusions; swollen aggregated mitochondria; loss of cristae.
Chatfield *et al.* (2015)	N.S.	2 months	Mild enchephalomyopathy, diffusion of the perirolandic white matter (n=1).	*HSD10* mutation[g] *c.740A>G* (p.N247S)	Loss of cristae; swollen vacuolated mitochondria.
Nozuma *et al.* (2015)	N.S.	32 and 76 years	Mitochondrial myopathy, limb weakness and	16 non-pathogenic	Accumulation of mitochondria, vacuolated mitochondria.

			general fatigue, high CK, myalgia (n=2).	mtDNA alterations[i]: Pathogenic N.S.	

*Age at biopsy for EM study
AV: Atrioventricular
CPEO: Chronic progressive external opthalmoplegia
IMF: Intermyofibrillar
KSS: Kearns-sayer syndrome
MELAS: Mitochondrial encephalomyopathy, lactic acidosis and stoke-like episodes
mtDNA: Mitochondrial DNA
MERRF: Myoclonic epilepsy and ragged red fibres
N.S.: Not specified
PCI: Paracrystalline inclusion
PEO: progressive external opthalmoplegia
RRF: Ragged-red fibers
SS: Subsarcolemmal
[1]: common large-scale deletion, 4977bp in length between two 13-bp direct repeats at positions *m.13447-13459* and *m.8470-8482.*
Genetic diagnostic methodology: [a]Restriction site mutation assay; [b]Southern blot, [c]PCR, [d]Sanger sequencing, [e]RFLP, [f]Solid phase mini sequencing, [g]Sequencing, [h]Next generation sequencing, [i]Not specified

References

Abu-Amero, K., H. Al-Dhalaan, S. Bohlega, A. Hellani, and R. Taylor. 2009. A patient with typical clinical features of mitochondrial encephalopathy, lactic acidosis and stroke-like episodes (MELAS) but without an obvious genetic cause: A case report. *Journal of Medical Case Reports*. 3 (no pagination).

Adachi, M., J. Torii, B.W. Volk, P. Briet, A. Wolintz, and L. Schneck. 1973. Electron microscopic and enzyme histochemical studies of cerebellum, ocular and skeletal muscles in chronic progressive ophthalmoplegia with cerebellar ataxia. *Acta Neuropathologica*. 23:300-312.

Afifi, A.K., M.Z.M. Ibrahim, R.A. Bergman, N.A. Haydar, J. Mire, N. Bahuth, and F. Kaylani. 1972. Morphologic features of hypermetabolic mitochondrial disease. A light microscopic, histochemical and electron microscopic study. *Journal of the Neurological Sciences*. 15:271-290.

Ali, N., C.E. Woodward, M. Sweeney, R. Phadke, J.L. Holton, J. Acheson, G.T. Plant, and F.D. Bremner. 2010. Pupillary dysfunction in an atypical case of mitochondrial myopathy with tubular aggregates. *Journal of neuro-ophthalmology : the official journal of the North American Neuro-Ophthalmology Society*. 30:153-156.

Angelini, C., P. Melacini, M.L. Valente, H. Reichmann, R. Carrozzo, M. Fanin, L. Vergani, G.M. Boffa, A. Martinuzzi, and G. Fasoli. 1993. Hypertrophic cardiomyopathy with mitochondrial myopathy - A new phenotype of complex II defect. *Japanese Heart Journal*. 34:63-77.

Arruda, W.O., L.F. Torres, A. Lombes, S. DiMauro, B.A. Cardoso, H.A. Teive, D. De Paola, and R.R. Seixas. 1990. Mitochondrial myopathy and myoclonic epilepsy. *Arquivos de neuro-psiquiatria*. 48:32-43.

Ascaso, F.J., E. Lopez-Gallardo, E. Del Prado, E. Ruiz-Pesini, and J. Montoya. 2010. Macular lesion resembling adult-onset vitelliform macular dystrophy in Kearns-Sayre syndrome with multiple mtDNA deletions. *Clinical and Experimental Ophthalmology*. 38:812-816.

Baric, I., K. Fumic, D. Petkovic Ramadza, W. Sperl, F.A. Zimmermann, D. Muacevic-Katanec, Z. Mitrovic, L. Pazanin, L. Cvitanovic Sojat, T. Kekez, Z. Reiner, and J.A. Mayr. 2013. Mitochondrial myopathy associated with a novel 5522G>A mutation in the mitochondrial tRNA(Trp) gene. *European journal of human genetics : EJHG*. 21:871-875.

Baskin, E., U. Selda Bayrakci, F. Alehan, H. Ozdemir, A. Oner, R. Horvath, V. Vega-Warner, F. Hildebrandt, and F. Ozaltin. 2011. Respiratory-chain deficiency presenting as diffuse mesangial sclerosis with NPHS3 mutation. *Pediatric nephrology (Berlin, Germany)*. 26:1157-1161.

Bastiaensen, L.A.K., H.H.J. Jaspar, and A.M. Stadhouders. 1979. Ophthalmoplegia-plus. *Documenta Ophthalmologica*. 46:365-380.

Black, J.T., D. Judge, L. Demers, and S. Gordon. 1975. Ragged-red fibers. A biochemical and morphological study. *Journal of the Neurological Sciences*. 26:479-488.

Blakely, E.L., A. Butterworth, R.D. Hadden, I. Bodi, L. He, R. McFarland, and R.W. Taylor. 2012. MPV17 mutation causes neuropathy and leukoencephalopathy with multiple mtDNA deletions in muscle. *Neuromuscular disorders : NMD*. 22:587-591.

Bostan, A., G. Glibert, B. Dachy, and B. Dan. 2012. Novel mutation in spacer region of POLG associated with ataxia neuropathy spectrum and gastroparesis. *Autonomic Neuroscience: Basic and Clinical*. 170:70-72.

Bresolin, N., M. Zeviani, E. Bonilla, R.H. Miller, R.W. Leech, S. Shanske, M. Nakagawa, and S. DiMauro. 1985. Fatal infantile cytochrome c oxidase deficiency: decrease of immunologically detectable enzyme in muscle. *Neurology*. 35:802-812.

Cardaioli, E., P.D. Pozzo, A. Cerase, F. Sicurelli, A. Malandrini, N.D. Stefano, M.L. Stromillo, C. Battisti, M.T. Dotti, and A. Federico. 2006. Rapidly progressive neurodegeneration in a case with the 7472insC mutation and the A7472C polymorphism in the mtDNA tRNAser(UCN) gene. *Neuromuscular Disorders*. 16:26-31.

Cardenas, J.F., and R.S. Amato. 2010. Compound heterozygous polymerase gamma gene mutation in a patient with alpers disease. *Seminars in Pediatric Neurology*. 17:62-64.

Carta, A., V. Carelli, T. D'Adda, F.N. Ross-Cisneros, and A.A. Sadun. 2005. Human extraocular muscles in mitochondrial diseases: comparing chronic progressive external ophthalmoplegia with Leber's hereditary optic neuropathy. *The British journal of ophthalmology*. 89:825-827.

Carta, A., T. D'Adda, F. Carrara, and M. Zeviani. 2000. Ultrastructural analysis of extraocular muscle in chronic progressive external ophthalmoplegia. *Archives of ophthalmology (Chicago, Ill. : 1960)*. 118:1441-1445.

Castro-Gago, M., A. Alonso, E. Pintos-Martínez, A. Beiras-Iglesias, Y. Campos, J. Arenas, M.I. Novo-Rodríguez, and J. Eirís-Puñal. 1999. Congenital hydranencephalic-hydrocephalic syndrome associated with mitochondrial dysfunction. *Journal of Child Neurology*. 14:131-135.

Cenacchi, G., P. Valentina, F. Marina, P. Elena, and A. Corrado. 2011. Comparison of muscle ultrastructure in myasthenia gravis with anti-MuSK and anti-AChR antibodies. *Journal of Neurology*. 258:746-752.

Chatfield, K.C., C.R. Coughlin, M.W. Friederich, R.C. Gallagher, J.R. Hesselberth, M.A. Lovell, R. Ofman, M.A. Swanson, J.A. Thomas, R.J.A. Wanders, E.P. Wartchow, and J.L.K. Van Hove. 2015. Mitochondrial energy failure in HSD10 disease is due to defective mtDNA transcript processing. *Mitochondrion*. 21:1-10.

Chen, T., C. Pu, Q. Shi, Q. Wang, L. Cong, J. Liu, H. Luo, L. Fei, W. Tang, and S. Yu. 2014. Chronic progressive external ophthalmoplegia with inflammatory myopathy. *International journal of clinical and experimental pathology*. 7:8887-8892.

Chi, C.S., H.F. Lee, C.R. Tsai, H.J. Lee, and L.H. Chen. 2010. Clinical manifestations in children with mitochondrial diseases. *Pediatric neurology*. 43:183-189.

Colombo, A., E. Merelli, P. Sola, P. Panzetti, D. Quaglino Jr, and C. Fornieri. 1988. Mitochondrial oculoskeletal myopathy: case report. *The Italian Journal of Neurological Sciences*. 9:385-389.

Conway, L.J., T.E. Robertson, J.J. McGill, and J.P. Hanson. 2011. MELAS syndrome in an Indigenous Australian woman. *Medical Journal of Australia*. 195:581-582.

Coquet, M., F. Degoul, A. Vital, M. Malgat, J.P. Mazat, C. Louvet-Giendaj, D. Fontan, F. Tison, M. Diry, and C. Marsac. 1993. Merrf family with 8344 mutation in tRNA (lys). Evidence of a mitochondrial vasculopathy in muscle biopsies. *Neuromuscular disorders : NMD*. 3:593-597.

Coulbault, L., D. Herlicoviez, F. Chapon, M.H. Read, M.J. Penniello, P. Reynier, G. Fayet, A. Lombès, P. Jauzac, and S. Allouche. 2005. A novel mutation in the mitochondrial tRNAAsn gene associated with a lethal disease. *Biochemical and Biophysical Research Communications*. 329:1152-1154.

Crosby, T.W., and S.M. Chou. 1974. "Ragged-red" fibers in Leigh's disease. *Neurology*. 24:49-54.

De Kremer, R.D., A. Paschini-Capra, S. Bacman, C. Argarana, G. Civallero, R.I. Kelley, N. Guelbert, A. Latini, I. Noher de Halac, A. Giner-Ayala, J. Johnston, R. Proujansky, I. Gonzalez, C. Depetris-Boldini, A. Oller-Ramirez, C. Angaroni, R.A. Theaux, E. Hliba, and E. Juaneda. 2001. Barth's syndrome-like disorder: a new phenotype with a maternally inherited A3243G substitution of mitochondrial DNA (MELAS mutation). *American journal of medical genetics*. 99:83-93.

DeBrosse, S., E.E. Ubogu, S. Yaniglos, M.O. Hassan, and R.J. Leigh. 2009. Dynamic properties of eye movements in mitochondrial chronic progressive external ophthalmoplegia. *Eye (London, England)*. 23:382-388.

Deschauer, M., H. Swalwell, M. Strauss, S. Zierz, and R.W. Taylor. 2006. Novel mitochondrial transfer RNA(Phe) gene mutation associated with late-onset neuromuscular disease. *Archives of neurology*. 63:902-905.

Dias-Tosta, E. 1988. Chronic progressive external ophthalmoplegia. II. A qualitative and quantitative electronmicroscopy study of skeletal muscles. *Arquivos de Neuro-Psiquiatria*. 46:143-155.

Dinopoulos, A., J. Smeitink, and H. ter Laak. 2005. Unusual features of mitochondrial degeneration in skeletal muscle of patients with nuclear complex I mutation. *Acta Neuropathol*. 110:199-202.

Enns, G.M., C.L. Hoppel, S.J. DeArmond, S. Schelley, N. Bass, K. Weisiger, D. Horoupian, and S. Packman. 2005. Relationship of primary mitochondrial respiratory chain dysfunction to fiber type abnormalities in skeletal muscle. *Clinical genetics*. 68:337-348.

Fagiolari, G., M. Sciacco, L. Chiveri, C. Lamperti, G.P. Comi, G. Scarlato, M. Moggio, and A. Prelle. 2002. Lack of apoptosis in patients with progressive external ophthalmoplegia and mutated adenine nucleotide translocator-1 gene. *Muscle and Nerve*. 26:265-269.

Federico, A., L. Manneschi, M. Meloni, C. Alessandrini, A.M. Bardelli, M.T. Dotti, and P. Sabatelli. 1988. Histochemical, ultrastructural and biochemical study of muscle mitochondria in Leber's hereditary optic atrophy. *Journal of Inherited Metabolic Disease*. 11:193-197.

Fillano, J.J., M.J. Goldenthal, C.H. Rhodes, and J. Marin-Garcia. 2002. Mitochondrial dysfunction in patients with hypotonia, epilepsy, autism, and developmental delay: HEADD syndrome. *J Child Neurol*. 17:435-439.

Fischer, J.C., W. Ruitenbeek, F.J.M. Gabreëls, A.J.M. Janssen, W.O. Renier, R.C.A. Sengers, A.M. Stadhouders, H.J. ter Laak, J.M.F. Trijbels, and J.H. Veerkamp. 1986. A mitochondrial encephalomyopathy: the first case with an established defect at the level of coenzyme Q. *European Journal of Pediatrics*. 144:441-444.

Fitzsimons, R.B., and H.D.D. Tyer. 1980. A study of a myopathy presenting as idiopathic scoliosis. Multicore disease or mitochondrial myopathy? *Journal of the Neurological Sciences*. 46:33-48.

Fukuhara, N., S. Tokiguchi, K. Shirakawa, and T. Tsubaki. 1980. Myoclonus epilepsy associated with ragged-red fibres (mitochondrial abnormalities): disease entity or a syndrome? Light-and electron-microscopic studies of two cases and review of literature. *Journal of the neurological sciences*. 47:117-133.

Gilbert, R.D., and M. Emms. 1996. Pearson's syndrome presenting with Fanconi syndrome. *Ultrastructural Pathology*. 20:473-475.

Gotz, A., P. Isohanni, B. Liljestrom, J. Rummukainen, K. Nikolajev, E. Herrgard, S. Marjavaara, and A. Suomalainen. 2012. Fatal neonatal lactic acidosis caused by a novel de novo mitochondrial G7453A tRNA-Serine ((UCN)) mutation. *Pediatr Res*. 72:90-94.

Güçer, Ş., B. Talim, E. Aşan, P. Korkusuz, S. Özen, Ş. Ünal, S.H. Kalkanoğlu, G. Kale, and M. Çağlar. 2005. Focal segmental glomerulosclerosis associated with mitochondrial cytopathy: Report of two cases with special emphasis on podocytes. *Pediatric and Developmental Pathology*. 8:710-717.

Haginoya, K., S. Miyabayashi, K. Iinuma, and K. Tada. 1990. Mosaicism of mitochondria in mitochondrial myopathy: an electronmicroscopic analysis of cytochrome c oxidase. *Acta Neuropathologica*. 80:642-648.

Han, Y., B. Deng, M. Liu, J. Jiang, S. Wu, and Y. Guan. 2010. Clinical and genetic study of a Chinese family with spinocerebellar ataxia type 7. *Neurology India*. 58:622-626.

Hayes, D.J., B.R.F. Lecky, D.N. Landon, J.A. Morgan-Hughes, and J.B. Clark. 1984. A new mitochondrial myopathy. Biochemical studies revealing a deficiency in the cytochrome b-c1 complex (complex III) of the respiratory chain. *Brain : a journal of neurology*. 107:1165-1177.

Heiman-Patterson, T.D., Z. Argov, J.M. Chavin, B. Kalman, H. Alder, S. DiMauro, W. Bank, and A.J. Tahmoush. 1997. Biochemical and genetic studies in a family with mitochondrial myopathy. *Muscle & nerve*. 20:1219-1224.

Herrero-Marti, N.M.D., T. Ayuso, M.T. Tunon, M.A. Martin, E. Ruiz-Pesini, and J. Montoya. 2010. A MELAS/MERRF phenotype associated with the mitochondrial DNA 5521G>A mutation. *Journal of Neurology, Neurosurgery and Psychiatry*. 81:471-472.

Higashikata, T., J. Koyama, H. Shimada, M. Yazaki, M. Owa, and S. Ikeda. 2001. An 80-year-old mitochondrial disease patient with A3243G tRNA(Leu(UUR)) gene presenting cardiac dysfunction as the main symptom. *Internal medicine (Tokyo, Japan)*. 40:405-408.

Holliday, P.L., A.R. Climie, J. Gilroy, and M.Z. Mahmud. 1983. Mitolchondrial myopathy and encephalopathy: Three cases—a deficiency of NADH-CoQ dehydrogenase? *Neurology*. 33:1619-1622.

Hopmann, D., A. Kivi, W. Stenzel, R. Ehret, and J. Mueller. 2014. Late onset myopathy and hyperkinetic movement disorder. *Clinical Neurophysiology*. 125:S209.

Hudgson, P., W.G. Bradley, and M. Jenkison. 1972. Familial "mitochondrial" myopathy. A myopathy associated with disordered oxidative metabolism in muscle fibres Part 1. Clinical, electrophysiological and pathological findings. *Journal of the Neurological Sciences*. 16:343-370.

Ionasescu, V.V., M. Hart, S. DiMauro, and C.T. Moraes. 1994. Clinical and morphologic features of a myopathy associated with a point mutation in the mitochondrial tRNA(Pro) gene. *Neurology*. 44:975-977.

Karpati, G., S. Carpenter, A.G. Engel, G. Watters, J. Allen, S. Rothman, G. Klassen, and O.A. Mamer. 1975. The syndrome of systemic carnitine deficiency. Clinical, morphologic, biochemical, and pathophysiologic features. *Neurology*. 25:16-24.

Karppa, M., R. Herva, A.R. Moslemi, A. Oldfors, S. Kakko, and K. Majamaa. 2005. Spectrum of myopathic findings in 50 patients with the 3243A>G mutation in mitochondrial DNA. *Brain : a journal of neurology*. 128:1861-1869.

Kendall, F.D. 2012. Mitochondrial disorders: Overview of diagnostic tools and new diagnostic trends. *Journal of Pediatric Biochemistry*. 2:193-203.

Kennaway, N.G., N.R.M. Buist, V.M. Darley–usmar, A. Papadimitriou, S. Dimauro, R.I. Kelley, R.A. Capaldi, N.K. Blank, and A. D'Agostino. 1984. Lactic acidosis and mitochondrial myopathy associated with deficiency of several components of complex III of the respiratory chain. *Pediatric Research*. 18:991-999.

Kim, D.S., D.S. Jung, K.H. Park, I.J. Kim, C.M. Kim, W.H. Lee, and S.K. Rho. 2002. Histochemical and molecular genetic study of MELAS and MERRF in Korean patients. *J Korean Med Sci*. 17:103-112.

Kim, G.W., S.M. Kim, I.N. Sunwoo, and J.G. Chi. 1991. Two cases of mitochondrial myopathy with predominant respiratory dysfunction. *Yonsei medical journal*. 32:184-189.

Kim, J.S., C.J. Kim, J.G. Chi, and H.J. Myung. 1989. Chronic progressive external ophthalmoplegia (CPEO) with 'ragged red fibers'. A case report. *Journal of Korean Medical Science*. 4:91-96.

Kim, S.H., and J.G. Chi. 1997. Characterization of a mitochondrial DNA deletion in patients with mitochondrial myopathy. *Molecules and cells*. 7:726-729.

Korenke, G.C., H.A.C.M. Bentlage, W. Ruitenbeek, R.C.A. Sengers, W. Sperl, J.M.F. Trijbels, F.J.M. Gabreels, F.A. Wijburg, V. Wiedermann, F. Hanefeld, U. Wendel, M. Reckmann, V. Griebel, and H. Wölk. 1990. Isolated and combined deficiencies of NADH dehydrogenase (complex I) in muscle tissue of children with mitochondrial myopathies. *European Journal of Pediatrics*. 150:104-108.

Kyriacou, K., A. Hadjisavvas, A. Zenios, R. Papacharalambous, and T. Kyriakides. 2005. Morphological methods in the diagnosis of mitochondrial encephalomyopathies: the role of electron microscopy. *Ultrastruct Pathol*. 29:169-174.

Kyriacou, K., C. Mikellidou, A. Hadjianastasiou, L. Middleton, A. Panousopoulos, and T. Kyriakides. 1999. Ultrastructural diagnosis of mitochondrial encephalomyopathies revisited. *Ultrastructural Pathology*. 23:163-170.

Kyriakides, T., A. Drousiotou, A. Panasopoulou, A. Hadjisavvas, A. Zenios, G.M. Hadjigeorgiou, and K. Kyriacou. 2003. A comparative morphological study in 33 cases of respiratory chain encephalomyopathies. *Acta Myologica*. 22:48-51.

Land, J.M., J.M. Hockaday, J.T. Hughes, and B.D. Ross. 1981. Childhood mitochondrial myopathy with ophthalmoplegia. *Journal of the Neurological Sciences*. 51:371-382.

Lang, T., N. Laver, M.B. Strominger, A. Witking, R. Pfannl, and J. Alroy. 2010. Morphological findings of extraocular myopathy with chronic progressive external ophthalmoplegia. *Ultrastruct Pathol*. 34:78-81.

Lehmann, J., J. Ziegan, G. Oertel, J. Lößner, and H.J. Kühn. 1986. Myopathy with mitochondrial abnormalities and rimmed vacuoles. *Acta Neuropathologica*. 70:86-90.

Letellier, T., M. Malgat, M. Coquet, B. Moretto, F. Parrot-Roulaud, and J.P. Mazat. 1992. Mitochondrial myopathy studies on permeabilized muscle fibers. *Pediatr Res*. 32:17-22.

Linda, S., I. Lund, T. Torbergsen, J. Aasly, S.I. Mellgren, O. Borud, and P. Monstad. 1992. Mitochondrial diseases and myopathies: A series of muscle biopsy specimens with ultrastructural changes in the mitochondria. *Ultrastructural Pathology*. 16:263-275.

Linden, G.J.v.d., and B.J.J. Ansink. 1979. A case of the Kearns - Shy syndrome. *Clinical Neurology and Neurosurgery*. 81:45-52.

Lopez, C.P., P.G. Rios, E.R. Infante, P. Carbonell, M. Hirano, and S. DiMauro. 2013. TK2 mutation presenting as indolent myopathy. *Neurology. Conference: 65th American Academy of Neurology Annual Meeting San Diego, CA United States. Conference Start*. 80.

Lu, J., and Y. Huang. 2013. Childhood mitochondrial encephalomyopathies: clinical course, diagnosis, neuroimaging findings, mtDNA mutations and outcome in six children. *Italian journal of pediatrics*. 39:60.

Makino, M., S. Horai, Y.I. Goto, and I. Nonaka. 2000. Mitochondrial DNA mutations in Leigh syndrome and their phylogenetic implications. *Journal of Human Genetics*. 45:69-75.

Marín-García, J., M.J. Goldenthal, L. Flores-Sarnat, and H.B. Sarnat. 2002. Severe mitochondrial cytopathy with complete A-V block, PEO, and mtDNA deletions. *Pediatric neurology*. 27:213-216.

McKelvie, P.A., J.B. Morley, E. Byrne, and S. Marzuki. 1991. Mitochondrial encephalomyopathies: A correlation between neuropathological findings and defects in mitochondrial DNA. *Journal of the Neurological Sciences*. 102:51-60.

McLeod, J.G., D.C. Baker, C.D. Shorey, and C.B. Kerr. 1975. Mitochondrial myopathy with multisystem abnormalities and normal ocular movements. *Journal of the Neurological Sciences*. 24:39-52.

Melberg, A., P.O. Lundberg, K.G. Henriksson, Y. Olsson, and E. Stålberg. 1996. Muscle-nerve involvement in autosomal dominant progressive external ophthalmoplegia with hypogonadism. *Muscle and Nerve*. 19:751-757.

Melegh, B., L. Seress, T. Bedekovics, G. Kispál, B. Sümegi, K. Trombitás, and K. Méhes. 1999. Muscle carnitine acetyltransferase and carnitine deficiency in a case of mitochondrial encephalomyopathy. *Journal of Inherited Metabolic Disease*. 22:827-838.

Mierau, G.W., R.W. Tyson, and C.L. Freehauf. 2004. Role of electron microscopy in the diagnosis of mitochondrial cytopathies. *Pediatric and developmental pathology : the official journal of the Society for Pediatric Pathology and the Paediatric Pathology Society*. 7:637-640.

Miles, L., B.L. Wong, A. Dinopoulos, P.J. Morehart, I.A. Hofmann, and K.E. Bove. 2006. Investigation of children for mitochondriopathy confirms need for strict patient selection, improved morphological criteria, and better laboratory methods. *Human pathology*. 37:173-184.

Mitsumoto, H., J.R. Aprille, S.H. Wray, R. Nemni, and W.G. Bradley. 1983. Chronic progressive external ophthalmoplegia (CEPO): Clinical, morphologic, and biochemical studies. *Neurology*. 33:452-461.

Mizusawa, H., M. Watanabe, I. Kanazawa, T. Nakanishi, M. Kobayashi, M. Tanaka, H. Suzuki, M. Nishikimi, and T. Ozawa. 1988. Familial mitochondrial myopathy associated with peripheral neuropathy: Partial deficiencies of complex I and complex IV. *Journal of the Neurological Sciences*. 86:171-184.

Modi, G., J.M. Heckman, and D. Saffer. 1992. Vitelliform macular degeneration associated with mitochondrial myopathy. *The British journal of ophthalmology*. 76:58-60.

Müller-Höcker, J., I. Paetzke, D. Pongratz, and G. Hübner. 1985. Mitochondrial myopathy with diffuse activation and focal deficiency of mitochondrial ATPase and carnitine deficiency. *Virchows Archiv B Cell Pathology Including Molecular Pathology*. 48:185-196.

Müller-Höcker, J., D. Pongratz, and G. Hübner. 1983. Focal deficiency of cytochrome-c-oxidase in skeletal muscle of patients with progressive external ophthalmoplegia - Cytochemical-fine-structural study. *Virchows Archiv A Pathological Anatomy and Histopathology*. 402:61-71.

Nevo, Y., D. Soffer, M. Kutai, N. Zelnik, A. Saada, J. Jossiphov, G. Messer, A. Shaag, E. Shahar, S. Harel, and O. Elpeleg. 2002. Clinical characteristics and muscle pathology in myopathic mitochondrial DNA depletion. *J Child Neurol*. 17:499-504.

Niebrój-Doblosz, I., B. Ryniewicz, A. Fidziańska, and B. Badurska. 1985. Lipid storage myopathy in Kearns-Sayre syndrome. *Neurology*. 35:1582-1586.

Nolte, K.W., S. Trepels-Kottek, D. Honnef, J. Weis, C.G. Bien, A. van Baalen, K. Ritter, B. Czermin, S. Rudnik-Schoneborn, N. Wagner, and M. Hausler. 2013. Early muscle and brain ultrastructural changes in polymerase gamma 1-related encephalomyopathy. *Neuropathology : official journal of the Japanese Society of Neuropathology*. 33:59-67.

Norby, S., P. Lestienne, I. Nelson, I.M. Nielsen, H. Schmalbruch, O. Sjo, and M. Warburg. 1994. Juvenile Kearns-Sayre syndrome initially misdiagnosed as a psychosomatic disorder. *Journal of medical genetics*. 31:45-50.

Nozuma, S., Y. Okamoto, I. Higuchi, J. Yuan, A. Hashiguchi, Y. Sakiyama, A. Yoshimura, Y. Higuchi, and H. Takashima. 2015. Clinical and electron microscopic findings in two patients with mitochondrial myopathy associated with episodic hyper-creatine kinase-emia. *Internal Medicine*. 54:3209-3214.

Patterson, K. 2004. Mitochondrial muscle pathology. *Pediatric and developmental pathology : the official journal of the Society for Pediatric Pathology and the Paediatric Pathology Society*. 7:629-632.

Paulus, W., A. Stevens, and W. Roggendorf. 1989. Mitochondrial encephalomyopathy with pilovacuolar inclusion or phenocopy with mitochondrial artefact? *Journal of Neurology*. 236:361-363.

Perry, M.S., and J.T. Sladky. 2008. Neuroradiologic findings in Sengers syndrome. *Pediatric neurology*. 39:113-115.

Pfeffer, G., P.J. Waters, J. Maguire, H.D. Vallance, V.A. Wong, and M.M. Mezei. 2012. Levator palpebrae biopsy and diagnosis of progressive external ophthalmoplegia. *The Canadian journal of neurological sciences. Le journal canadien des sciences neurologiques*. 39:520-524.

Polimeno, L., R. Rossi, M. Mastrodonato, M. Montagnani, D. Piscitelli, B. Pesetti, L. De Benedictis, B. Girardi, L. Resta, A. Napoli, and A. Francavilla. 2013. Augmenter of liver regeneration, a protective factor against ROS-induced oxidative damage in muscle tissue of mitochondrial myopathy affected patients. *The international journal of biochemistry & cell biology*. 45:2410-2419.

Prick, M.J.J., F.J. M. Gabreëls, J.M.F. Trijbels, A.J.M. Janssen, R. le Coultre, K. van Dam, H.H. J. Jaspar, E. J. Ebels, and A.A.W.O. de Coul. 1983. Progressive poliodystrophy (alpers') disease) with a defect in cytochromeaa3 in muscle: a report of two unrelated patients. *Clinical Neurology and Neurosurgery*. 85:57-70.

Pronicki, M., E. Matyja, D. Piekutowska-Abramczuk, T. Szymanska-Debinska, A. Karkucinska-Wieckowska, E. Karczmarewicz, W. Grajkowska, T. Kmiec, E. Popowska, and J. Sykut-Cegielska. 2008. Light and electron microscopy characteristics of the muscle of patients with SURF1 gene mutations associated with Leigh disease. *Journal of clinical pathology*. 61:460-466.

Pulkes, T., D. Liolitsa, L.H. Eunson, M. Rose, I.P. Nelson, S. Rahman, J. Poulton, D.R. Marchington, D.N. Landon, A.G. Debono, J.A. Morgan-Hughes, and M.G. Hanna. 2005. New phenotypic diversity associated with the mitochondrial tRNA(SerUCN) gene mutation. *Neuromuscular disorders : NMD*. 15:364-371.

Reichmann, H., R. Gold, B. Meurers, M. Naumann, P. Seibel, U. Walter, and T. Klopstock. 1993. Progression of myopathology in Kearns-Sayre syndrome: a morphological follow-up study. *Acta Neuropathologica*. 85:679-681.

Rivner, M.H., M. Shamsnia, T.R. Swift, J. Trefz, R.A. Roesel, A.L. Carter, W. Yanamura, and F.A. Hommes. 1989. Kearns-Sayre syndrome and complex II deficiency. *Neurology*. 39:693-696.

Roefs, A.M., P.J. Waters, G.R.W. Moore, and P.J. Dolman. 2012. Orbicularis oculi muscle biopsies for mitochondrial DNA analysis in suspected mitochondrial myopathy. *British Journal of Ophthalmology*. 96:1296-1299.

Rollins, S., R.A. Prayson, J.T. McMahon, and B.H. Cohen. 2001. Diagnostic yield of muscle biopsy in patients with clinical evidence of mitochondrial cytopathy. *American Journal of Clinical Pathology*. 116:326-330.

Saneto, R.P., and A. Bouldin. 2006. A boy with muscle weakness, hypercarbia, and the mitochondrial DNA A3243G mutation. *Journal of Child Neurology*. 21:77-79.

Sarnat, H.B., G. Machin, H.Z. Darwish, and S.Z. Rubin. 1983. Mitochondrial myopathy of cerebro-hepato-renal (Zellweger) syndrome. *Canadian Journal of Neurological Sciences*. 10:170-177.

Schaaf, C.P., M. Blazo, R.A. Lewis, R.E. Tonini, H. Takei, J. Wang, L.J. Wong, and F. Scaglia. 2011. Early-onset severe neuromuscular phenotype associated with compound heterozygosity for OPA1 mutations. *Molecular genetics and metabolism*. 103:383-387.

Schapira, A.H.V., J.M. Cooper, J.A. Morgan-Hughes, D.N. Landon, and J.B. Clark. 1990. Mitochondrial myopathy with a defect of mitochondrial-protein transport. *New England Journal of Medicine*. 323:37-42.

Sengers, R.C.A., J.C. Fischer, J.M.F. Trijbels, W. Ruitenbeek, A.M. Stadhouders, H.J. ter Laak, and H.H.J. Jaspar. 1983. A mitochondrial myopathy with a defective respiratory chain and carnitine deficiency. *European Journal of Pediatrics*. 140:332-337.

Shah, A.J., V. Sahgal, G. Muschler, V. Subramani, and H. Singh. 1982. Morphogenesis of the mitochondrial alterations in muscle diseases. *J Neurol Sci*. 55:25-37.

Shibasaki, H., T. Santa, and Y. Kuroiwa. 1973. Late onset mitochondrial myopathy. *Journal of the Neurological Sciences*. 18:301-310.

Siriwardena, K., N. Mackay, V. Levandovskiy, S. Blaser, J. Raiman, P.F. Kantor, C. Ackerley, B.H. Robinson, A. Schulze, and J.M. Cameron. 2013. Mitochondrial citrate synthase crystals: novel finding in Sengers syndrome caused by acylglycerol kinase (AGK) mutations. *Molecular genetics and metabolism*. 108:40-50.

Smith, M.L., X.Y. Hua, D.L. Marsden, D. Liu, N.G. Kennaway, K.Y. Ngo, and R.H. Haas. 1997. Diabetes and mitochondrial encephalomyopathy with lactic acidosis and stroke-like episodes (MELAS): radiolabeled polymerase chain reaction is necessary for accurate detection of low percentages of mutation. *The Journal of clinical endocrinology and metabolism*. 82:2826-2831.

Stadhouders, A.M., P.H.K. Jap, H.P. Winkler, H.M. Eppenberger, and T. Wallimann. 1994. Mitochondrial creatine kinase: A major constituent of pathological inclusions seen in mitochondrial myopathies. *Proceedings of the National Academy of Sciences of the United States of America*. 91:5089-5093.

Stadhouders, A.M., and R.C.A. Sengers. 1987. Morphological observations in skeletal muscle from patients with a mitochondrial myopathy. *Journal of Inherited Metabolic Disease*. 10:62-80.

Stenqvist, L., A. Paetau, L. Valanne, A. Suomalainen, and H. Pihko. 2005. A juvenile case of MELAS with T3271C mitochondrial DNA mutation. *Pediatr Res*. 58:258-262.

Sumegi, B., B. Melegh, K. Adamovich, and K. Trombitas. 1990. Cytochrome oxidase deficiency affecting the structure of the myofibre and the shape of mitochondrial cristae membrane. *Clinica Chimica Acta*. 192:9-18.

Suzuki, T., J. Koizumi, H. Shiraishi, K. Ofuku, M. Sasaki, T. Hori, and N. Oskoshi. 1989. Psychiatric disturbance in mitochondrial encephalomyopathy. *Journal of neurology, neurosurgery, and psychiatry*. 52:920-922.

Takahashi, S., Y. Makita, J. Oki, A. Miyamoto, J. Yanagawa, E. Naito, Y. Goto, and A. Okuno. 1998. De novo mtDNA nt 8993 (T-->G) mutation resulting in Leigh syndrome. *American journal of human genetics*. 62:717-719.

Takeda, S., E. Ohama, and F. Ikuta. 1990. Involvement of extraocular muscle in mitochondrial encephalomyopathy. *Acta Neuropathologica*. 80:118-122.

Telerman-Toppet, N., D. Biarent, J.M. Bouton, L. de Meirleir, C. Elmer, S. Noel, E. Vamos, and S. DiMauro. 1992. Fatal cytochrome c oxidase-deficient myopathy of infancy associated with mtDNA depletion. Differential involvement of skeletal muscle and cultured fibroblasts. *Journal of Inherited Metabolic Disease*. 15:323-326.

Thajeb, P., Y.S. Ma, C.Y. Tzen, C.K. Chuang, T.Y. Wu, S.C. Chen, and Y.H. Wei. 2006. Oculopharyngeal somatic myopathy in a patient with a novel large-scale 3,399 bp deletion and a homoplasmic T5814C transition of the mitochondrial DNA. *Clin Neurol Neurosurg*. 108:407-410.

Toscano, A., M. Santoro, G. Vita, P. Girlanda, S. Sinicropi, M.C. Fazio, A. Mazzeo, C. Rodolico, M. Aguennouz, S. Bartolone, L. Bet, G.P. Comi, and C. Messina. 1996. Late-onset mitochondrial neuromyopathy: An age-related phenomenon? *Archives of Gerontology and Geriatrics*. 22:577-583.

Uusimaa, J., S. Finnila, L. Vainionpaa, M. Karppa, R. Herva, H. Rantala, I.E. Hassinen, and K. Majamaa. 2003. A mutation in mitochondrial DNA-encoded cytochrome c oxidase II gene in a child with Alpers-Huttenlocher-like disease. *Pediatrics*. 111:e262-268.

van Domburg, P.H., A.A. Gabreels-Festen, F.J. Gabreels, R. de Coo, W. Ruitenbeek, P. Wesseling, and H. ter Laak. 1996. Mitochondrial cytopathy presenting as hereditary sensory neuropathy with progressive external ophthalmoplegia, ataxia and fatal myoclonic epileptic status. *Brain : a journal of neurology*. 119 (Pt 3):997-1010.

van Ekeren, G.J., A.M. Stadhouders, G.J.M. Egberink, R.C.A. Sengers, O. Daniëls, and K. Kubat. 1987. Hereditary mitochondrial hypertrophic cardiomyopathy with mitochondrial myopathy of skeletal muscle, congenital cataract and lactic acidosis. *Virchows Archiv A Pathological Anatomy and Histopathology*. 412:47-52.

van Wijngaarden, G.K., J. Bethlem, A.E. Meijer, W.C. Hulsmann, and C.A. Feltkamp. 1967. Skeletal muscle disease with abnormal mitochondria. *Brain : a journal of neurology*. 90:577-592.

Vogel, H. 2001. Mitochondrial myopathies and the role of the pathologist in the molecular era. *Journal of neuropathology and experimental neurology*. 60:217-227.

Wabbels, B., J.A. Schroeder, B. Voll, H. Siegmund, and B. Lorenz. 2007. Electron microscopic findings in levator muscle biopsies of patients with isolated congenital or acquired ptosis. *Graefe's archive for clinical and experimental ophthalmology = Albrecht von Graefes Archiv fur klinische und experimentelle Ophthalmologie*. 245:1533-1541.

Wada, H., M. Woo, H. Nishio, S. Nagaki, H. Yanagawa, A. Imamura, S. Yokoyama, C. Ohbayashi, M. Matsuo, H. Itoh, and H. Nakamura. 1996. Vascular involvement in benign infantile mitochondrial myopathy caused by reversible cytochrome c oxidase deficiency. *Brain & development*. 18:263-268.

Wang, L.C., W.T. Lee, W.Y. Tsai, Y.K. Tsau, and Y.Z. Shen. 2000. Mitochondrial cytopathy combined with Fanconi's syndrome. *Pediatric neurology*. 22:403-406.

Yau, E.K.C., K.Y. Chan, K.M. Au, T.C. Chow, and Y.W. Chan. 2009. A novel mitochondrial DNA deletion in a Chinese girl with Kearns-Sayre syndrome. *Hong Kong Medical Journal*. 15:374-377.

Yerdelen, D., F. Koc, and Z. Koc. 2008. Delayed diagnosis of Kearns-Sayre syndrome in a 38-year-old male patient: a case report. *The International journal of neuroscience*. 118:267-275.

Yuan, J.H., Y. Sakiyama, I. Higuchi, Y. Inamori, Y. Higuchi, A. Hashiguchi, K. Higashi, A. Yoshimura, and H. Takashima. 2013. Mitochondrial myopathy with autophagic vacuoles in patients with the m.8344A>G mutation. *Journal of clinical pathology*. 66:659-664.

Zeharia, A., N. Fischel-Ghodsian, K. Casas, Y. Bykhovskaya, H. Tamari, D. Lev, M. Mimouni, and T. Lerman-Sagie. 2005. Mitochondrial myopathy, sideroblastic anemia, and lactic acidosis: An automosal recessive syndrome in persian jews caused by a mutation in the PUS1 gene. *Journal of Child Neurology*. 20:449-452.

Zhao, D., Z. Wang, D. Hong, W. Zhang, and Y. Yuan. 2013. Chronic progressive external ophthalmoplegia coexistent with motor neuron disease in a patient with a novel large-scale mitochondrial DNA deletion. *Clinical Neurology and Neurosurgery*. 115:1490-1492.
